# Combined Use of Genome-Wide Association Data and Correlation Networks Unravels Key Regulators of Primary Metabolism in *Arabidopsis thaliana*

**DOI:** 10.1371/journal.pgen.1006363

**Published:** 2016-10-19

**Authors:** Si Wu, Saleh Alseekh, Álvaro Cuadros-Inostroza, Corina M. Fusari, Marek Mutwil, Rik Kooke, Joost B. Keurentjes, Alisdair R. Fernie, Lothar Willmitzer, Yariv Brotman

**Affiliations:** 1 Max Planck Institute of Molecular Plant Physiology, Potsdam-Golm, Germany; 2 MetaSysX GmbH, Potsdam-Golm, Germany; 3 Laboratory of Genetics, Wageningen University, Wageningen, the Netherlands; 4 Department of Life Sciences, Ben Gurion University of the Negev, Beersheva, Israel; University of California, UNITED STATES

## Abstract

Plant primary metabolism is a highly coordinated, central, and complex network of biochemical processes regulated at both the genetic and post-translational levels. The genetic basis of this network can be explored by analyzing the metabolic composition of genetically diverse genotypes in a given plant species. Here, we report an integrative strategy combining quantitative genetic mapping and metabolite‒transcript correlation networks to identify functional associations between genes and primary metabolites in *Arabidopsis thaliana*. Genome-wide association study (GWAS) was used to identify metabolic quantitative trait loci (mQTL). Correlation networks built using metabolite and transcript data derived from a previously published time-course stress study yielded metabolite‒transcript correlations identified by covariation. Finally, results obtained in this study were compared with mQTL previously described. We applied a statistical framework to test and compare the performance of different single methods (network approach and quantitative genetics methods, representing the two orthogonal approaches combined in our strategy) with that of the combined strategy. We show that the combined strategy has improved performance manifested by increased sensitivity and accuracy. This combined strategy allowed the identification of 92 candidate associations between structural genes and primary metabolites, which not only included previously well-characterized gene‒metabolite associations, but also revealed novel associations. Using loss-of-function mutants, we validated two of the novel associations with genes involved in tyrosine degradation and in β-alanine metabolism. In conclusion, we demonstrate that applying our integrative strategy to the largely untapped resource of metabolite–transcript associations can facilitate the discovery of novel metabolite-related genes. This integrative strategy is not limited to *A*. *thaliana*, but generally applicable to other plant species.

## Author Summary

Primary metabolites are key elements in plant growth and development. Our partial understanding of their biosynthesis and regulation derives mostly from biochemical and genetic modification experiments. The recent generation of large-scale genome-wide data, along with the advances in mass-spectrometry techniques, allows us to treat metabolite levels as quantitative traits and to link them to genomic information, resulting in the identification of so-called metabolic quantitative trait loci (mQTL). These mQTL contribute to the discovery of new biosynthetic and regulatory elements that control the plant's metabolic landscape. Low mapping resolution, however, normally limits discovery to one causal gene per locus. Here, we utilize a complementary strategy to support the identification of casual genes by genetic mapping. We measured metabolite levels in 314 *A*. *thaliana* accessions, then used genome-wide association mapping to identify mQTL. We next used previously published results from a time-course stress study to construct metabolite‒transcript correlation networks. Integrating data from both approaches enabled us to select candidate genes linked to specific metabolites. We finally validated two of the novel gene‒metabolite associations using knockout lines. We demonstrated that by using an integrative strategy, we can validate previously characterized gene–metabolite associations, and most importantly, identify novel associations between metabolites and genes. The combined quantitative genetics and metabolite‒transcript networks that we present here can be applied to other organisms and fields of research.

## Introduction

Plants produce a large array of structurally and biologically diverse metabolites. Largely due to the missing underlying biochemistry, the genes encoding metabolite-related enzymes or regulatory proteins are known for only a fraction of the metabolites. With the development of metabolomic and genomic tools, alternative approaches have been successfully applied to identify genes encoding enzymes involved in specific biochemical pathways [1–6].

Metabolite levels can be used as quantitative traits, and quantitative trait locus (QTL) mapping of metabolite levels using structured populations facilitates the identification of the genomic regions associated with the metabolic variation [7–9]. However, given the relatively low resolution reached using this approach [10], the cloning of single causal genes has rarely been achieved. Genome-wide association studies (GWAS), due to the presence of many more meiotic events present in natural populations during historical recombination, allow a more refined QTL resolution [11, 12]. However, the limitation of GWAS, especially in self-mating biological systems such as *Arabidopsis thaliana*, lies not only in the generation of false positive genotype‒phenotype associations because of the confounding effects of population structure [13, 14], but also in the poor resolution reached if associated SNPs are found in extensive islands of haplotypes in linkage disequilibrium (LD) [15–17]. Epistasis and lack of natural variation can also result in a high false-negative rate, wherein loci with previous experimental validation for specific traits are not found in GWAS [17, 18]. In order to take advantage of both resources, a growing number of recent reports have successfully combined mQTL from bi-parental segregating populations and natural populations to elucidate the biochemical nature of metabolite traits [19–21]. Due to limited segregating allelic diversity in bi-parental segregating populations such as recombinant inbred lines (RIL) and introgression lines (IL), the validation of GWAS results is not possible in every case [22]. The combination of both GWAS and bi-parental segregating populations, however, is advantageous in reducing the false-positive associations in GWAS due to the fact that in many cases, even after population structure correction, some individuals might be more related to each other than individuals are related on average [23, 24].

Aside from genetic evidence, the integration of additional forms of genome-scale data, such as metabolite and transcript data, has been applied to detect metabolite‒gene correlations and to largely reduce false-positive correlations [6, 25–27]. To date, network analysis has mainly focused on correlations between transcripts and transcripts (i.e. co-expression networks) [28], and correlations between metabolites and metabolites (i.e. metabolic networks) [29]. The study of metabolite‒transcript correlations is yet to be fully explored. Detection and elucidation of metabolite‒transcript correlations can yield important clues regarding the consequences of altered environmental conditions on metabolite levels in organismal systems [30]. Although a few pioneering investigations have tried to apply this integrative strategy [6, 19, 31–35], the power of combined results from the two orthogonal approaches, i.e. quantitative genetics and metabolite‒transcript networks, for the elucidation of the genetic architecture of metabolite traits has not been fully exploited. Based on first principles, the overlap of results obtained using these two approaches in parallel should increase their statistical confidence.

**Fig 1:**
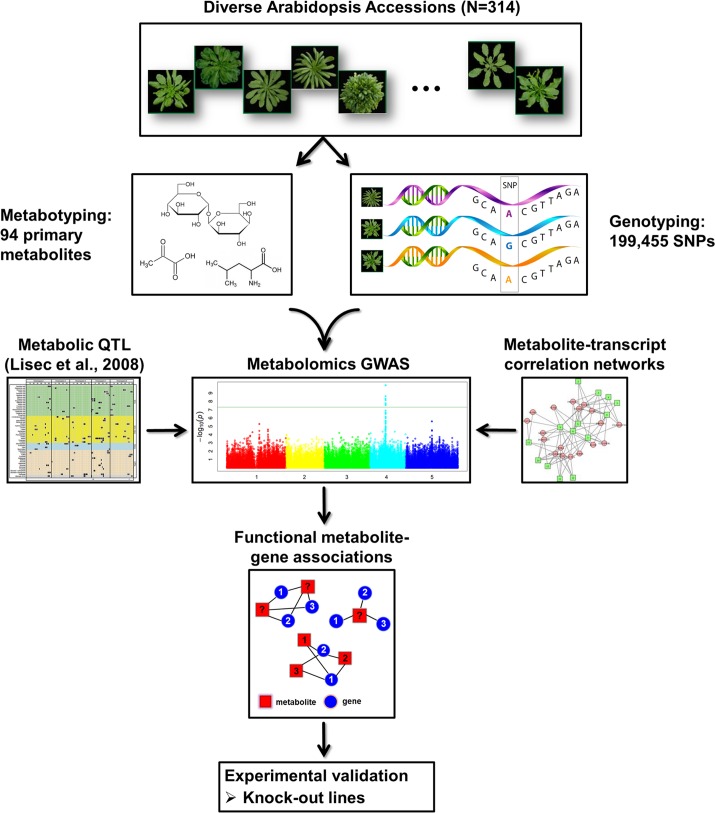
Data integration workflow for the systematic detection of candidate metabolite‒transcript associations in primary metabolism in *A*. *thaliana*. We combined high-throughput gas chromatography‒mass spectrometry (GC‒MS)-based metabolomics and genotyping data [65, 66] in genome-wide association studies (GWAS). GWAS results were then compared with 157 identified quantitative trait loci (QTL) [8] and metabolite‒transcript correlation information from a time-course experiment that recorded the plants’ responses to changing light and/or temperature [36]. The obtained hypotheses were then subject to experimental verification by transgenic methods.

In order to test this hypothesis, we analyzed 94 primary metabolites in a densely genotyped collection of 314 natural *A*. *thaliana* accessions, and used these metabolite levels as phenotypic traits to conduct a GWAS with 200K single nucleotide polymorphisms (SNPs). The resulting metabolite‒gene associations from the GWAS were compared and validated with mQTL which had been described before using two *A*. *thaliana* populations (429 RILs and 97 ILs) [8]. In parallel, metabolite‒transcript correlation networks were constructed based on reported transcriptome and metabolome levels of *A*. *thaliana* as a function of changing environments [36]. Correlations identified between metabolites and transcripts were applied as an additional and independently derived filtering criterion to further support identified metabolite‒gene associations. Furthermore, we applied a statistical analysis framework to test and compare the performance of all single methods (GWAS, RIL, IL, and network analysis) with that of the combined strategy by using *precision*, *recall* and *F-measure*. The results indicate that the combined strategy (the strategy to predict genes supported by network analysis and at least one mapping approach) exhibits an overall better performance as compared to the single methods, boasting increased sensitivity and accuracy. Using this integrative strategy (**Fig 1**), 92 main metabolite‒gene associations were identified. The validity of the approach was confirmed by analyzing two loss-of-function mutants for two novel genes. In conclusion, this study serves as a proof of concept, demonstrating that by integrating two orthogonal approaches, novel metabolite‒gene associations can be obtained with a robust statistical significance.

## Results

### Metabolic profiling by GC‒MS

**Fig 2:**
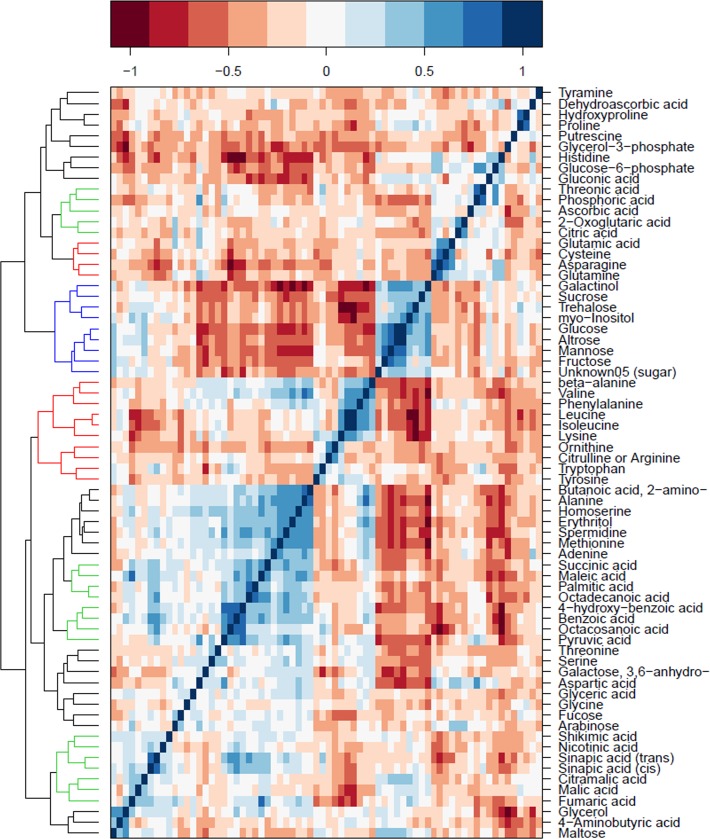
Correlation pattern among measured primary metabolites. Pairwise Pearson correlations (*r*^*2*^) are calculated between each metabolite across all 314 accessions. Primary metabolites are ordered using Ward clustering on pairwise dissimilarity. The clustered metabolites are highlighted with colors according to the chemical classes of primary metabolites: amino acids, organic acids and sugars are colored red, green and blue, respectively.

Information about the *A*. *thaliana* accessions used in this study is provided in **S1 Table**. 94 metabolic features, comprising 26 amino acids, 23 organic acids, 17 sugars, three amines, four other metabolites with known, and 21 with unknown, chemical structure, were reproducibly detected in rosette material of 314 *A*. *thaliana* accessions. Metabolite ID, name, classification, and quantification mass used for the following data analysis are shown in **S2 Table**. Normalized metabolite data across 314 accessions are shown in **S1 Dataset**. Those metabolites belonging to one functional class were highly correlated, demonstrated by the fact that ten amino acids, nine sugars, and some organic acids were clustered together, respectively (**Fig 2**).

### Genome-wide association study of the *A*. *thaliana* primary metabolome

The metabolic profiles of the accessions revealed that 37.8% of all annotated metabolites were associated with at least one locus at a genome-wide significance level of *p* ≤ 5.01 × 10^−6^ (LOD = 5.3), calculated by a mixed linear model. This model includes principal components as fixed effects to account for population structure (commonly called the “Q” matrix) [37], and a kinship matrix (commonly called the “K” matrix) [38]. In order to test how well the model used in GWAS accounts for population structure and familial relatedness across the accessions, we generated quantile‒quantile (QQ) plots for all 94 metabolite traits. We observed that the majority of points in the QQ plot lay on the diagonal line for all the metabolite traits, indicating that spurious associations due to population structure and familial relatedness were largely corrected. The SNPs in the upper right section of the QQ plot deviating from the diagonal were most likely associated with the metabolite traits in the study. The QQ plots for the metabolite traits further discussed here are shown in **S1 Fig**. In total, 117 distinct SNP‒trait associations, resulting in 617 gene‒metabolite-trait associations, were identified (**S3 and S4 Tables**). In the following, two representative examples of these associations will be described in more detail.

### Example 1: GWAS confirms existing annotation of a gene: The homoserine kinase gene

A strong association (*p* = 4.11 × 10^−6^, LOD = 5.39) between SNP m59466 at the AT2G17265 locus and the metabolite trait homoserine was detected. Gene AT2G17265 encodes a homoserine kinase (*HSK*) that catalyzes the chemical reaction with the substrate L-homoserine to produce *O*-phospho-L-homoserine (HserP), a compound at the branching point of methionine and threonine biosynthesis [39]. A loss-of-function mutant of this gene results in higher levels of the amino acid homoserine [40], which is in line with the observation described here.

### Example 2: Haplotype analysis strongly suggests the tyramine decarboxylase gene as the causative locus modulating tyramine levels

**Fig 3:**
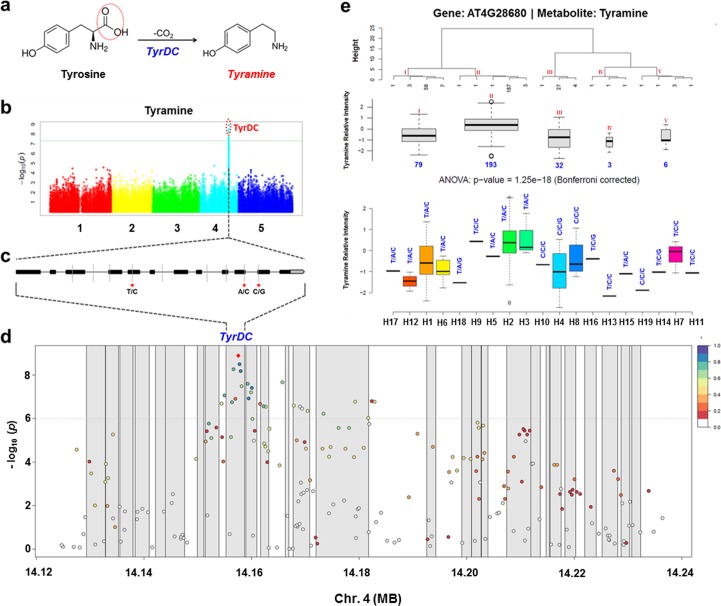
An exemplary association found by GWAS between the metabolite trait tyramine and *TyrDC*. (a) Decarboxylic reaction that tyramine is involved in with the candidate gene product *TyrDC*. (b) Manhattan plot for the metabolite trait tyramine and significant association signals. *P* values are shown on a log_10_ scale, the x-axis shows the physical positions on five chromosomes in *A*. *thaliana*. Significantly associated SNPs in *TyrDC* are highlighted in the red circle (c) Gene model of *TyrDC*. Filled black boxes represent coding sequence. The light gray vertical lines mark the polymorphic sites identified by high-throughput genotyping [65, 66] and the stars represent the proposed functional sites. (d) Linkage disequilibrium (LD) plot for the locus associated to tyramine levels on a zoomed-in Manhattan plot. The x-axis shows the physical positions in this LD block on chromosome 4, the y-axis shows the significance levels with *p* values on a log_10_ scale. Each gray block denotes a gene in the locus to which tyramine mapped. Each dot serves as one SNP marker and the lead SNP (with highest LOD) is shown with red diamond. Imputation revealed several closely located SNPs in strong LD (*r*^*2*^) with the lead SNP. (e) Haplotype analysis for nine SNPs genotyped in candidate gene *TyrDC*. Haplotypes were further clustered according to their similarities in five groups based on Ward's minimum variance method (upper panel). Box-plots show the tyramine intensity for these 5 different clusters (middle panel; box width represents number of accessions in the cluster) and for the various haplotypes (bottom panel; the three potential functional SNP variations for each haplotype are shown above each box). One-way ANOVA was applied to detect differences between cluster means, followed by Bonferroni correction for multiple comparisons (*p* < 0.01).

Tyramine was significantly associated with SNP m154079 (*p* = 1.28 × 10^−9^, LOD = 8.89) (**Fig 3B**). Lead SNP m154079 and other significantly associated SNPs, are located in locus *TyrDC* (L-tyrosine decarboxylase 1, AT4G28680), which was reported to encode a stress-induced tyrosine decarboxylase [41]. This enzyme catalyzes a dicarboxylic reaction on tyrosine to release CO_2_ and produce tyramine (**Fig 3A**). There are nine SNP markers in this gene identified by high-throughput genotyping (**Fig 3C**). Among these nine SNPs, three SNPs leading to changes in the amino-acid sequence are located in the fifth, tenth, and eleventh exon, respectively. The first polymorphism variant (T/C, m154077) results in a serine-to-proline substitution, the second SNP variant (A/C, m154081) causes a serine-to-arginine exchange, and the third SNP (C/G, m154082) brings about a more subtle substitution, from serine to threonine (**Fig 3C**). Linkage disequilibrium (LD) analysis of the mapped genomic region for the tyramine trait revealed that the three exonic SNPs (m154077, m154081, and m154082) are highly and significantly linked with the lead SNP m154079 (r^2^ > 0.75, *p* < 0.001) (**Fig 3D**). This finding suggests that they are likely to constitute the functional variation underlying this association. However, it is still difficult to completely exclude other variants surrounding this region. Therefore, we took the nine SNP markers in *TyrDC* to conduct haplotype analysis for the accessions. These nine SNPs give rise to 19 possible haplotypes, eight of them being informative haplotypes defined by more than two accessions within a haplotype. The haplotypes can be further classified into five main clusters according to the haplotype sequence similarities. Cluster II (H2, H3, H5, H9, and H18) presents significantly higher levels of tyramine than Cluster I (H1, H6, H12, and H17), Cluster III (H4, H10 and H18), as well as two other minor clusters (Cluster IV and V) (**Fig 3E**). Taken together, both the associated SNPs and the haplotype analysis support *TyrDC* as a candidate gene controlling tyramine levels.

### GWAS comparison with metabolic QTL from RIL and IL populations

One of the main aims of this study is to discover true and novel metabolite‒gene associations involved in *A*. *thaliana* primary metabolism by integrating various quantitative genetics and network approaches. To this end, we compared the GWAS obtained in this study with results reported previously based on the analysis of two *A*. *thaliana* bi-parental populations: 429 RIL and 97 IL derived from accessions Col-0 and C24 [8]. Out of the 40 metabolite traits described in the RIL dataset, 32 overlap with those of the GWAS, whereas 50 metabolites overlap between the GWAS and the IL data (*cf*. **S5** and **S6 Tables** for the mQTL identified in RIL and IL in [8], respectively). It has been described that in many cases the Bonferroni threshold is too stringent for quantitative gene identification [42]. We therefore decided to test the performance of the GWAS when different LOD thresholds were applied based on the four reference gene lists (RGL1– RGL 4) derived from KEGG metabolic pathways (see Materials and Methods, section “Procedure setup for determining method performance”; *cf*. **S7 Table**). GWAS performance using various LOD thresholds was evaluated by three statistics: *precision*, *recall* and *F-measure*. These three parameters, as well as the number of correctly predicted metabolites across all tested GWAS LOD thresholds (from 2.0 to 5.3) were recorded (**S8 Table)**. The measureable values for these four statistics increased with lower thresholds, but were not changed with LOD thresholds lower than 3.0 (**S8 Table**). Additionally, we tested the *metabolite-wise precision* for each metabolite when applying LOD thresholds ranging from 3.0 to 5.3. As shown in **S2 Fig**, the *metabolite-wise precision* was very low when applying relatively low LOD thresholds ranging from 3.0 to 4.0, implying that the chance of finding true functional related genes from a relatively large mapped locus is very low. LOD threshold 4.5 was selected for further integration with other methods, because it can balance well the trade-off between obtaining more correctly predicted metabolite traits and discovering the causal genes for metabolite traits more precisely. Comparison between different datasets was conducted using both the significant LOD threshold after Bonferroni correction (LOD = 5.3) and the suggestive LOD threshold (LOD = 4.5).

Common loci obtained by comparing QTL results from the GWAS, RIL, and IL datasets using the two GWAS LOD thresholds mentioned above are listed in **Table 1** and **S9 Table**. One example we would like to point out is the QTL detected for nicotinic acid, located on chromosome 5, with 41507 bp, supported by GWAS, RIL, and IL results together.

**Table 1:** Common loci verified by GWAS, RIL, and IL datasets (using GWAS LOD ≥ 5.3)

Trait	Chr	Left border of the locus (bp)	Right border of the locus (bp)	Number of genes in the locus	Confirmed by GWAS	Confirmed by RIL	Confirmed by IL
Nicotinic acid	5	4746332	4787839	10	T	T	T
Fructose	2	16970258	17012067	12	T	T	F
Leucine	4	8231017	8322201	30	T	T	F
Glutamic acid	1	2743761	2788447	11	T	F	T
Gluconic acid	3	9052982	9095537	10	T	F	T
Lysine	3	464279	521747	24	T	F	T
4-Aminobutyric acid	5	26854022	26883430	9	T	F	T

### Network analysis

Quantitative genetic analysis establishes the association between a locus/gene and a trait (here: metabolite) by testing the co-occurrence between trait variants and genetic markers. As an orthogonal, albeit still statistics-based approach, we decided to test the associations of metabolites with transcripts resulting from metabolite‒transcript correlation networks for their power to identify candidate genes involved in the synthesis and/or degradation of a given metabolite. Though this approach has been successfully used in many instances with secondary metabolites [25–27, 43, 44], the comparable investigation of primary metabolites has not been fully explored. Metabolite and transcript data were obtained from a previously published study from our group, in which the metabolomic and transcriptomic responses of *A*. *thaliana* towards eight environmental conditions differing in temperature and light intensity were recorded at a high kinetic time-resolved resolution [36]. Significantly changed metabolites across 23 time points in each condition at a significance level of 0.05 after multiple correction, together with all 15,089 transcripts, were used to construct condition-specific networks (eight individual networks in total). The numbers of primary metabolites and transcripts, as well as the statistically significant Pearson Correlation Coefficient (PCC) thresholds derived from permutation test for the individual networks, are shown in **S10 Table**. Multiple metabolite‒transcript correlations shared across different conditions were detected, suggesting conserved associations, 219 of them being maintained across all eight conditions (**S11 Table**). These highly robust correlations found between transcripts and primary metabolites indicate conserved/tight regulation in *A*. *thaliana*.

In order to test the likelihood of these correlations to be functionally significant, all metabolite‒transcript correlations detected by network analysis were compared with the GWAS. The common associations supported by both GWAS and network analysis under the two GWAS LOD thresholds are presented in **S12** and **S13 Tables**, respectively. In the following, we will describe some exemplary results in more detail. Temperature- and light-stress treatments were abbreviated as follows: (i) 4°C and darkness (4-D), (ii) 21°C and darkness (21-D), (iii) 32°C and darkness (32-D), (iv) 4°C and normal light (4-L), (v) 21°C and low light (21-LL), (vi) 21°C and normal light (21-L), (vii) 21°C and high light (21-HL), and (viii) 32°C and normal light (32-L).

Network data revealed a conserved and significant correlation between *SPMS* (spermidine synthase 3, AT5G53120) and β-alanine. For six conditions, high PCCs were observed (21-L, –0.61; 21-D, –0.75; 4-L, –0.78; 4-D, –0.87; 32-D, –0.64; 21-LL, –0.86). Furthermore, this association is in agreement with the GWAS data. *SPMS* is annotated as encoding a novel spermine synthase and is a paralog of previously characterized spermidine synthases, *SPDS1* and *SPDS2* [45, 46]. The protein that *SPMS* encodes can catalyze the reaction from spermine to spermidine, and thus fuel the subsequent two steps in β-alanine biosynthesis.

A robust link between tyrosine and *TAT7* (tyrosine aminotransferase 7, AT5G53970) was observed in five out of eight condition-specific networks (the PCCs observed were: 4-L, 0.68; 21-LL, –0.65; 21-L, –0.69; 21-HL, –0.57; 32-D, 0.67). *TAT7* encodes a tyrosine aminotransferase as proven by both loss-of-function mutants and an *in vitro* recombinant protein assay, whereby it was suggested that *TAT7* is a tyrosine-specific aminotransferase not involved in tyrosine biosynthesis, but rather in the utilization of tyrosine for other metabolic pathways, e.g. tocopherol biosynthesis [47]. Levels of tyrosine, as a central primary metabolite, can be influenced by many factors. Its profiles observed for the five environmental conditions indicated that temperature may be the more influential element for tyrosine content rather than light intensity (**S3 Fig**). The correlation between tyrosine and *TAT7* is also supported by the RIL dataset.

**Fig 4:**
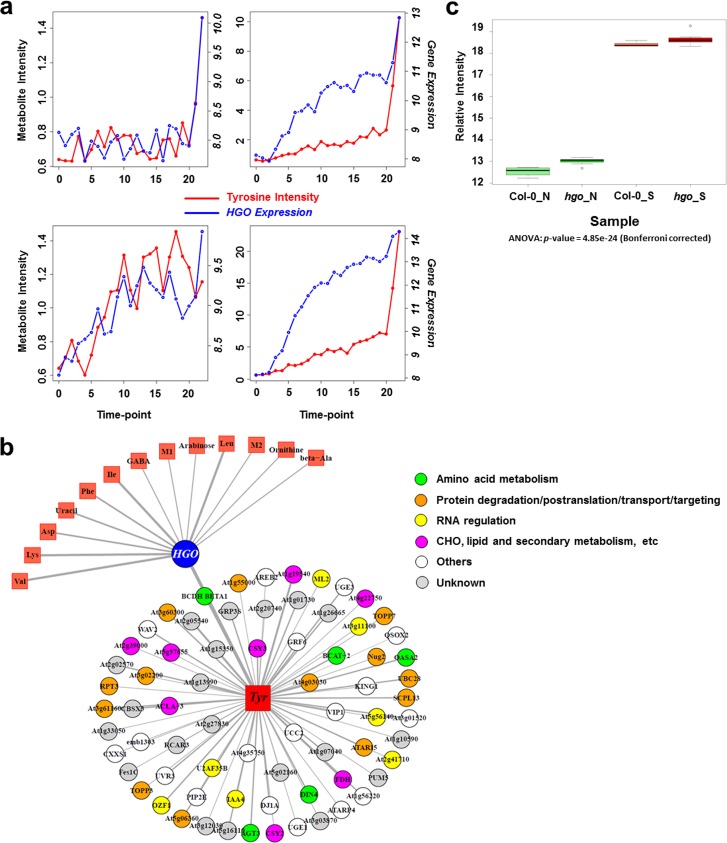
Association between tyrosine and *HGO*. (a) Profiles of tyrosine and *HGO* across 23 time points in four conditions (4-D, 21-D, 21-LL, and 32-D). Red and blue lines represent metabolite intensity and gene expression, respectively. (b) Merged network by combining the four condition-specific networks (4-D, 21-D, 21-LL, and 32-D). Node colors in the network stand for the functional classes to which transcripts belong. The width of edge in the network represents the number of conditions that a certain association between two nodes (corresponding metabolite and transcript) shares. In order to represent the most robust associations with tyrosine, only transcripts that are connected with tyrosine in all four conditions, and metabolites that are connected with *HGO* in at least two conditions, are displayed in this zoom-in merged transcript‒metabolite correlation network. (c) Box-plot of tyrosine intensity in wide-type (Col-0) plants and *HGO* mutant plants under normal and stress conditions (32-D). Tyrosine intensity is log_2_ transformed.

Another strong correlation discovered by the network analysis was between tyrosine and *HGO* (homogentisate 1, 2-dioxygenase, AT5G54080), displaying high positive correlations in three darkness conditions independent of temperature, and in another low-light stress condition: 4-D, 0.85; 21-D, 0.78; 21-LL, 0.74; 32-D, 0.73. The profiles of tyrosine and *HGO* across 23 time points in these four conditions are shown in **Fig 4A**. *HGO* is reported to encode a homogentisate 1,2-dioxygenase that can convert homogentisate to malylacetoacetate, and is likely to be involved in tyrosine degradation [48]. A merged network was constructed by combining the four condition-specific networks in 4-D, 21-D, 21-LL and 32-D stress conditions (**Fig 4B**). In order to represent the most robust correlations with tyrosine, only transcripts that are connected with tyrosine in all four conditions and metabolites that are connected with *HGO* in at least two conditions are displayed in this zoom-in merged metabolite‒transcript correlation network (**Fig 4B**). The merged network shows that the majority of associated transcripts belong to functional groups encoding amino-acid metabolism and protein degradation/post-translation/transport/targeting proteins, which is in line with the metabolic pathway for tyrosine. Again, the link between tyrosine and *HGO* is also supported by the RIL dataset.

### Comparison of the performance of individual and combined methods

**Fig 5:**
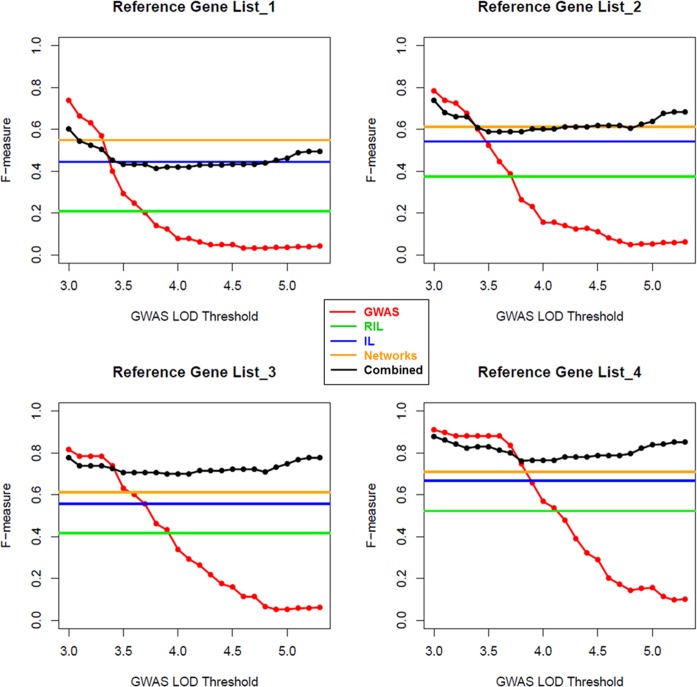
*F-measure* comparison between the single methods (GWAS, RIL, IL, and network analysis) and the combined strategy using different LOD thresholds based on the four reference gene lists.

A major goal of this study was to test the power of integrating results obtained by various quantitative genetics and network approaches for increased robustness and sensitivity. The performance of each single method and of the combined strategy (network analysis and at least one mapping approach) was tested by calculating *precision*, *recall* and *F-measure*, widely applied as scoring metrics in pattern recognition and information retrieval [49], based on different LOD thresholds ranging from 3.0 to 5.3. As a comparison set, we built four reference gene lists (RGL1, RGL 2, RGL 3, and RGL 4) for all the metabolites shown in the different datasets based on KEGG metabolic pathway [50] (see Materials and Methods, section “Procedure setup for determining method performance”; *cf*. **S7 Table)**. As shown in **S4 Fig** for *precision*, **S5 Fig** for *recall*, and **Fig 5** for *F-measure*, the combined strategy performs better than any other single method based on RGL2 (LOD ranging from 4.5 to 5.3), RGL3 (LOD ranging from 3.5 to 5.3) and RGL4 (LOD ranging from 3.8 to 5.3), except in the case of RGL1, in which the network approach performs better than the combined strategy (LOD ranging from 3.4 to 5.3), indicating that the network approach is superior to the combined strategy with regard to providing information about genes directly linked to the metabolite (neighbor transcripts). It is however important to note that the combined strategy performed better when applying the two selected LOD thresholds (significant threshold 5.3 and suggestive threshold 4.5) in this study based on RGL2 to RGL4.

In order to test whether the combined strategy has a better prediction ability of true associations as compared to random methods, we applied a randomization test in which we shuffled the related genes for all the annotated metabolites in the combined dataset, and obtained the permuted *F-measure* by comparing the shuffled related gene list with the four reference gene lists. After 10,000 iterations, the actual *F-measure* was compared with the permuted *F-measure* 10,000 times and an empirical *p*-value was estimated. **Table 2** shows the actual *F-measure*, permuted *F-measure*, and *p*-values when applying LOD thresholds 5.3 and 4.5. The results suggest that all the actual *F-measures* are significantly higher than the permuted ones, which means that the combined strategy using both significant and suggestive LOD thresholds performs significantly better than the randomized method.

**Table 2:** Permutation test of *F-measure* for the combined strategy with 10,000 iterations

Reference Gene List	LOD = 5.3			LOD = 4.5		
	Actual *F-measure*	Permuted *F-measure*	*P*-value	Actual *F-measure*	Permuted *F-measure*	*P*-value
RGL1	0.494	0.301 ± 0.055	5.00E-04	0.433	0.272±0.049	0.0007
RGL2	0.682	0.494 ± 0.052	3.00E-04	0.619	0.448±0.047	2.00E-04
RGL3	0.776	0.606 ± 0.048	4.00E-04	0.722	0.551±0.043	1.00E-04
RGL4	0.85	0.758 ± 0.039	0.008	0.788	0.678±0.036	0.002

The *metabolite-wise precision* is another important determinant parameter allowing us to compare the performance of different methods. Therefore, the *metabolite-wise precision* was calculated and compared across all the individual methods and the combined strategy. The comparison between methods for *metabolite-wise precision* based on all four reference gene lists and applying both significant and suggestive LOD thresholds (5.3 and 4.5) is shown in **S6** and **S7 Figs.** When applying LOD threshold 4.5, the *metabolite-wise precision* of the combined strategy is significantly higher than that of any other single methods based on RGL3 and RGL4 (combined strategy and network analysis: *p*-values are 0.029 and 0.050 based on RGL3 and RGL4, respectively), and showing the highest trend in the combined strategy based on RGL2. When using LOD threshold 5.3, the *metabolite-wise precision* of the combined strategy shows a trend higher than any other single method's based on RGL3 and RGL4. Overall, the results indicate that the combined strategy of integrating quantitative genetics and network analysis can largely improve the power of detection of true metabolite‒gene associations involved in *A*. *thaliana* primary metabolism.

### Candidate gene identification

**Fig 6:**
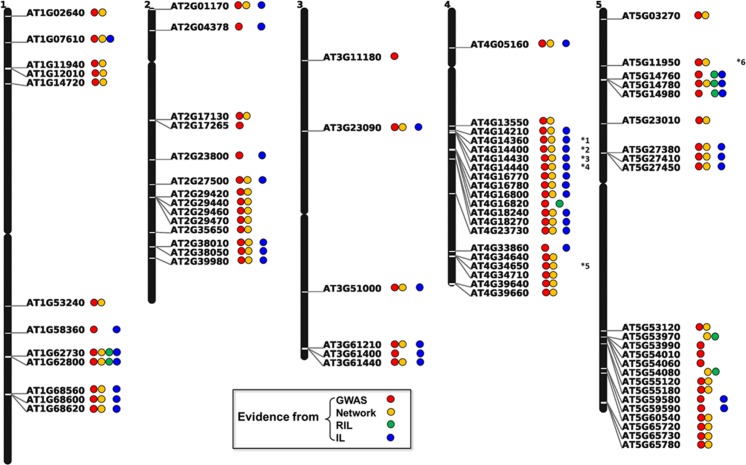
Chromosomal distribution of key metabolism-related candidate genes. Colored circles after each AGI code represent the approaches supporting each gene-metabolite association (red, blue, green and purple represent GWAS, network analysis, RIL, and IL, respectively). Asterisks with numbers after circles mean that the certain gene is associated with more than one specific metabolite. *1: associated with leucine,isoleucine, trehalose; *2 and *3: leucine, isoleucine, trehalose, phenylalanine and malic acid; *4: leucine, isoleucine, trehalose and phenylalanine; *5: galactinol and trehalose; *6: valine, leucine and isoleucine.

All associations between genes and primary metabolites detected by GWAS were cross-validated with the results from network analysis and from metabolic QTL results from RIL and IL populations using the two GWAS LOD thresholds described above. All associations supported by the four datasets are summarized in **S14** and **S15 Tables**, showing the overall comparison based on the two GWAS LOD thresholds evaluated. **Fig 6** represents the overall chromosomal distribution of 76 selected candidate genes in 92 main associations resulting from this study. Among them, 86 associations are supported by at least two of the approaches. One chromosomal hotspot supported by GWAS, network analysis, and QTL from IL population becomes immediately evident. It is located on chromosome 4, from 8231017 bp to 8366653 bp, and was previously reported to be related to biomass, resistance to a broad range of pathogens from different phyla [51], and to general metabolic activity [8]. Additional detailed information for candidate associations discussed in the text is listed in **Table 3**.

**Table 3:** Detailed information of candidate associations discussed in the text, selected by integrating the results from GWAS, network analysis, RIL, and IL

Metabolite	Gene	LOD	GWAS check	Networks check	RIL check	IL check	PCC ^*a*^								Annotation	Reference
							21L	21D	4L	4D	32L	32D	21LL	21HL		
Glycine	AT1G62800	4.77	T	T	T	T	-0.66	0	-0.73	0	0	0	0	0	aspartate aminotransferase 4	[52]
Nicotinic acid	AT5G14780	6.16	T	T	T	T	0	0.79	0	0	0	0	0	0	formate dehydrogenase	[53]
Nicotinic acid	AT5G14760	6.16	T	F	T	T	NA	NA	NA	NA	NA	NA	NA	NA	L-aspartate oxidase	[54]
beta-alanine	AT4G39660	5.16	T	T	F	F	0.85	0.82	0	0	0	0.61	0.65	0	alanine:glyoxylate aminotransferase 2	[55]
beta-alanine	AT5G53120	4.51	T	T	F	F	-0.61	-0.75	-0.79	-0.87	0	-0.64	-0.86	0	spermidine synthase 3	[45, 46]
Homoserine	AT2G17265	5.30	T	F	F	F	NA	NA	NA	NA	NA	NA	NA	NA	homoserine kinase	[40]
Tyrosine	AT5G54080	NA	F	T	T	F	0	0.78	NA	0.85	NA	0.73	0.74	NA	Encodes a homogentisate 1,2-dioxygenase that can convert homogentisate to malylacetoacetate and is likely to be involved in tyrosine catabolism	[48]
Tyrosine	AT5G53970	NA	F	T	T	F	-0.69	0	0.68	0	0	0.67	-0.65	-0.57	encodes tyrosine aminotransferase, strongly induced upon aging and coronatine treatment	[47]
Tyramine	AT4G28680	8.89	T	F	F	F	NA	NA	NA	NA	NA	NA	NA	NA	encode a stress-induced tyrosine decarboxylase	[41]

^*a*^ Value zero in PCC means that the actual absolute correlations are below the statistical PCC threshold and are rounded to 0. NA means that this association is not found in network analysis.

### Experimental validation

Validation of all associations disclosed is beyond the scope of this study. As a proof of concept, we focused on two promising candidate genes to experimentally validate our strategy and results. The first candidate gene is *HGO* (AT5G54080), associated with tyrosine in our analysis (**Fig 4A** and **4B**). Although the function of *HGO* was partly elucidated [48], genetic evidence based on mutant analysis to explore its metabolic roles in *A*. *thaliana* is still lacking. Therefore, a knockout line (SALK_027807) for *HGO* was grown in parallel with wild-type Col-0 plants under control (21-L) and stress (32-D) conditions (due to tyrosine showing dramatic accumulation in 32-D, the latter was chosen as the representative stress condition; see **S8 Fig**), whereupon both lines were subjected to GC‒MS metabolomic analysis. As evident from **Fig 4C**, tyrosine increased in both Col-0 and *hgo* plants under 32-D condition as compared to normal condition, in agreement with our previous report [36]. More importantly, however, we observed that tyrosine levels in the *hgo* mutant were significantly higher as compared to wild-type plants under normal condition (*p* = 0.002), and had increasing trends in the *hgo* mutant plants under stress conditions (32-D) (**Fig 4C**). These results are in line with the involvement of *HGO* in tyrosine degradation and confirm the usefulness of integrating information from network analysis and quantitative genetics approaches.

**Fig 7:**
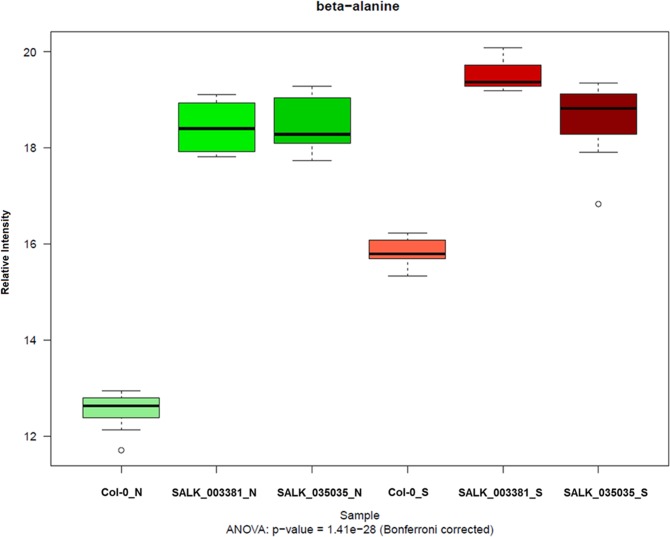
Functional assignment for candidate association between β-alanine and *AGT2*. Box-plot of β-alanine intensity in control plants (Col-0) and two independent knockout plants under normal (represented with “N”) and 32°C + darkness stress (represented with “S”) conditions. Significance levels among groups are evaluated by ANOVA followed by Bonferroni correction. Subsequent pair-wise comparison was conducted by Tukey HSD tests.

The second example concerns the gene *AGT2* (alanine:glyoxylate aminotransferase 2, AT4G39660). The association between *AGT2* and β-alanine is supported by both GWAS and network analysis. In the networks, this association displays high positive correlations under four conditions (21-D, 0.818; 21-LL, 0.649; 21-L, 0.849; 32-D, 0.613), representing very robust correlations between *AGT2* and β-alanine. In the GWAS, β-alanine mapped to a locus spanning 41 kb on chromosome 4. We considered three candidate genes encoding metabolic enzymes enclosed in this locus (AT4G39640, gamma-glutamyl transpeptidase 1, *GGT1*; AT4G39650, gamma-glutamyl transpeptidase 2, *GGT2*; AT4G39660, *AGT2*). *AGT2* is the only one supported also by network analysis, for which reason we selected it as the most promising candidate gene related to β-alanine. There are seven SNP markers in *AGT2*, five of them showing significant associations with β-alanine. Notably, one of the SNPs (m160527, position 18406944 bp on chromosome 4) can result in amino-acid substitution from proline (non-polar) to serine (polar) with the nucleotide variant from cytosine (C) to thymine (T). This suggests that SNP m160527 could be the causative SNP in *AGT2*. Based on sequence homology, *AGT2* was annotated as a putative alanine:glyoxylate aminotransferase. An attempt to functionally characterize *AGT2*, using an *in vitro* enzymatic assay, did not identify the enzyme as an alanine aminotransferase [55]. To date, the function of *AGT2* still remains unknown. Recently, Wen *et al* [34] also found that the close homolog of *AGT2* in maize (ZM01G05170) strongly mapped to β-alanine; this finding was further validated by their linkage analysis and eQTL (expression QTL) results. Therefore, we conducted a phylogenetic analysis on *AGT2* and its homologs from *A*. *thaliana* and from other plant species to explore the evolutionary history of this gene in plant taxa (**S9 Fig**). The first feature detected is the presence of at least two clusters, including sequences from both monocots and dicots, confirming that *AGT2* belongs to a multigene family. Interestingly, *AGT2* clustered together with the maize sequence ZM01G05170 reported by Wen *et al* [34], indicating that *AGT2* is the strict ortholog to the characterized enzyme in maize. In order to test for the role of *AGT2* in β-alanine metabolism, two independent loss-of-function lines (SALK_003381 and SALK_035035) for *AGT2*, plus wild-type plants, were grown under normal (21-L) and stress (32-D) conditions (32-D was selected as a representative stress condition because β-alanine strongly accumulated under this stress; see **S10 Fig**). β-alanine significantly increased in Col-0 plants under stress condition comparing with plants grown under control condition (*p* = 8.15E-13) (**Fig 7**), in agreement with previous observations [36]. More importantly, however, both KO plants displayed a very strong increase in β-alanine independent of the growth condition (**Fig 7**) (statistical significance levels by pair-wise comparison: SALK_003381_N & Col-0_N: 8.15E-13; SALK_035035_N & Col-0_N: 8.15E-13; SALK_003381_S & Col-0_S: 1.50E-12; SALK_035035_S & Col-0_S: 1.60E-12). These results thus suggest that *AGT2* is involved in β-alanine metabolism, reinforcing the utility in combining network and quantitative genetics analyses.

## Discussion

Metabolites are the terminal products of cellular regulatory processes, and their levels can be regarded as the ultimate responses of biological systems to environmental changes in a given genetic background, and thus serve as a link between subtle genotypes and visible phenotypes [56]. The genetic regulation of primary metabolites (essential for the viability of the cell) and secondary metabolites (required for the viability of the organism in the environment) is different. This derives from the fact that secondary metabolites are highly specific for particular genotypes, while primary metabolites are synthesized through common pathways and influenced by multiple and complicated factors [57]. Here, a GWAS strongly suggests polygenic regulation of primary metabolism in *A*. *thaliana*, owing to the fact that the individual metabolite traits mapped to multiple loci (each primary metabolite was mapped to 1.4 and 3.3 loci on average when applying the significant/suggestive LOD thresholds 5.3 and 4.5, respectively), which is in agreement with previous studies [32, 58]. The centrality and complexity of primary metabolism in *A*. *thaliana* makes it difficult to detect the true genetic‒metabolic relationships by a single method [6].

Within this study, we integrated GWAS based on a collection panel of 314 natural *A*. *thaliana* ecotypes, metabolite–transcript correlation network analysis for eight different environmental conditions based on data in [36], and mQTL results from two structured populations (RIL and IL; [8]). In order to test the validity of the combination of the two orthogonal approaches (quantitative genetics and network analysis) in comparison to each single method, we generated a statistical framework using four reference gene lists based on KEGG metabolic pathways (Materials and Methods, section “Procedure setup for determining method performance”). The performance of the different methods was evaluated and compared by *precision*, *recall* and *F-measure*, widely applied in pattern recognition and information retrieval [49]. We observed improved performance of the combined strategy (the strategy to predict genes supported by at least one mapping approach and network analysis) based on three out of four reference gene lists we applied (**S4** and **S5 Figs** and **Fig 5**). Although the combined strategy did not perform better than network approach based on RGL1, this indicates that network analysis outperforms the quantitative genetics methods in detecting enzymes directly linked to a given metabolite. Still, the combined strategy exhibited an overall better performance. Furthermore, the performance of the combined strategy was confirmed by permutation test (**Table 2**). Taken together, the statistical framework that we applied here illustrates that the combined strategy increases the sensitivity and robustness of candidate gene discovery.

Using the resulting metabolite–transcript associations, we identified connections between primary metabolites and structural genes that were previously reported to take part in the biosynthesis of the respective metabolites. For instance, the association between homoserine and AT2G17265 (*HSK*) supported by GWAS *per se* [40]; nicotinic acid and AT5G14760 (L-aspartate oxidase, *AO*) supported by GWAS, RIL, and IL results [54]; glycine and AT1G62800 (aspartate aminotransferase 4, *Asp4*) [52], nicotinic acid and AT5G14780 (formate dehydrogenase, *FDH*) [53] supported by all four datasets, illustrating the validity and feasibility of our combined strategy.

**Fig 8:**
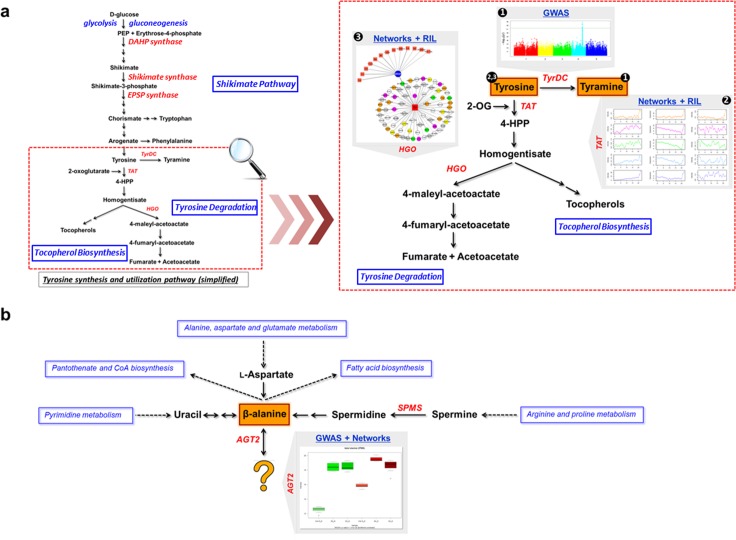
Exemplary candidate associations detected by the integrative strategy with GWAS, network analysis and mQTL from RIL and IL datasets. (a) Illustrated candidate genes and related discovery approaches in tyrosine synthesis and utilization pathway. (b) Illustrated candidate genes and related discovery approaches in β-alanine metabolism. *TyrDC*: tyrosine decarboxylase; *TAT*: tyrosine aminotransferase; *HGO*: homogentisate 1,2-dioxygenase; *SPMS*: spermine synthase.

Our integrative strategy offers a valuable tool not only for addressing previously reported primary metabolite‒gene associations, but also for discovering novel and under-explored candidate associations/genes involved in the regulation of *A*. *thaliana* primary metabolism. We found a strong association between tyramine and *TyrDC* (AT4G28680) in GWAS (**Fig 3B**). Analysis of SNPs leading to amino-acid substitution (**Fig 3C**), LD analysis (**Fig 3D**), and haplotype analysis (**Fig 3E**) supported *TyrDC* as the most prominent candidate gene for the metabolic trait tyramine. *TyrDC* was previously shown by enzymatic assay to encode a protein that catalyzes the conversion of tyrosine to tyramine [41]; our GWAS further provides genetic evidence for the gene annotation. Another two candidate genes that are also involved in tyrosine metabolism were discovered by network analysis, both of them being supported by the RIL dataset as well (**Fig 8A**). *TAT7* (AT5G53970), encoding a tyrosine aminotransferase whose products are 4-hydroxyphenylpyruvate (*4-HPP*) and L-glutamate [47], is linked to tyrosine in five conditions from the network analysis. *HGO*, previously shown to convert homogentisate to malylacetoacetate using *in vitro* enzymatic assays [48], is connected with tyrosine in four conditions from our network analysis. Using knockout lines, we further verified the function of *HGO* (**Fig 4C**) in tyrosine degradation. With the current knowledge on tyrosine synthesis and catabolism pathway, we could simultaneously identify three key genes in tyrosine degradation (**Fig 8A**). The detection of all these three critical genes manifests the strength of the integrative strategy based on the guilt-by-association principle [59].

We observed the strong correlation between tyrosine and *TAT7* in five conditions, showing negative correlations in three of them (21-LL, 21-L, and 21-HL; the common feature is 21°C), and two positive correlations in the stress conditions 4-L and 32-D (**S3 Fig**). One of the possible explanations for the flip of correlations for the same metabolite–transcript pair is that metabolic reactions, especially in primary metabolism, are regulated on different levels, and the metabolic fluxes are constantly changing when plants are exposed to various environmental stresses. It seems that a feedback loop regulation might control *TAT7* expression in a temperature-dependent manner. This is not always reflected in the actual tyrosine levels under different physiological conditions.

In the present study, we could identify two candidate genes (*SPMS*, AT5G53120 and *AGT2*, AT4G39660) involved in the β-alanine metabolic pathway, both supported by GWAS and network analysis (**Fig 8B**). In plants, three predicted pathways for β-alanine biosynthesis have been reported, including uracil degradation, polyamine oxidation, and propionate catabolism, but only the last enzyme in the uracil degradation pathway was studied in detail [60], leaving β-alanine metabolism in plants largely unexplored. The first candidate gene we identified is *SPMS*, reported to catalyze the conversion from spermine to spermidine by elongation of the polyamine chain [45, 61]. Notably, β-alanine can be produced by spermidine within the subsequent two reaction steps. Although *SPMS* has already been well characterized before, this example clearly demonstrates the power of the integrative strategy for detecting biochemically relevant associations between genes and metabolites that are not directly linked in a pathway (**Fig 8B**). We also identified a strong association between β-alanine and *AGT2*. Based on sequence homology, *AGT2* was annotated as a putative alanine:glyoxylate aminotransferase. Plant leaf peroxisomes are hypothesized to contain at least four aminotransferase activities, including Ser:glyoxylate aminotransferase (*SGT*), Glu:glyoxylate aminotransferase (*GGT*), Ala:glyoxylate aminotransferase (*AGT*), and Asp:glyoxylate aminotransferase (*AspAT*) [62, 63]. Animals possess two structurally distinct types of AGTs: *AGT1* and *AGT2*. Previous kinetic analysis of *A*. *thaliana AGT1* suggested that this protein mainly uses the substrates Ser and glyoxylate with SGT activity, while the function of *AGT2* remained obscure [55, 64]. We further tested this association using two independent knockout lines of *AGT2*. Both lines showed remarkable accumulation of β-alanine in comparison with wide-type plants, both in control and in stress conditions (**Fig 7**), supporting the association between β-alanine and *AGT2*. In *A*. *thaliana*, *AGT2* shows sequence homology to *AGT3* (AT2G38400) and *PYD4* (AT3G08860). Interestingly, *PYD4* is predicted to have β-alanine aminotransferase activity. Additionally, in maize, β-alanine mapped to a genetic locus harboring the homolog gene (ZM01G05170) of *AGT2*, which was further supported by linkage analysis and eQTL results [34]. In our phylogenetic analysis (**S9 Fig**), *AGT2* clustered together with its maize homolog ZM01G05170 reported by Wen *et al* [34], suggesting that both genes maintain the same function. *PYD4* (AT3G08860) clustered in a separate branch among sequences from other dicots before the speciation event (**S9 Fig**). Taking all the above evidence together with our findings using network analysis, GWAS and analysis of knockout lines, we can conclude that *AGT2* might be involved in β-alanine metabolism, but its decisive role as a β-alanine aminotransferase still needs to be confirmed by biochemical assays. It seems that *AGT2*, *PYD4* and *AGT3*, together with the maize homolog (ZM01G05170), are part of a large gene family of β-alanine aminotransferases, conserved both in monocot and dicot plants (**S9 Fig**).

Nowadays, GWAS is steadily becoming a common practice to identify the underlying genetic loci determining a plethora of phenotypic traits, but causal-gene identification still remains an obstacle. To overcome this, we present here a strategy based on the combined use of GWAS, metabolite–transcript correlation network analysis, and linkage mapping using structured populations, facilitating candidate association selection and providing functional and biological insight into *A*. *thaliana* primary metabolism. We demonstrate, using statistical analysis, that the combined strategy outperforms the single methods. Based on hypotheses generated by this comprehensive strategy, the functions of two novel genes were validated by transgenic methods. Our results illustrate that the integrative strategy described here offers an invaluable tool for advancing our knowledge of *A*. *thaliana* primary metabolism, a tool that can be applied to other plant species for functional elucidation of unknown genes. To our best knowledge, it is the first report to apply this combined strategy with all the above potent sources to cross-validate and prioritize candidate associations involved in *A*. *thaliana* primary metabolism.

## Materials and Methods

### Plant materials

#### Natural population and growth condition

A previously described collection of 314 natural *A*. *thaliana* accessions was used to measure primary metabolites for GWAS with existing SNP data [65, 66]. Seeds were sown on filter paper with demineralized water and stratified at 4°C in darkness for five days to break dormancy. Seeds were then transferred to a culture room (16 h LD, 24°C) for 42 h to induce seed germination. Each accession was transplanted onto wet Rockwool blocks of 4×4 cm in a climate chamber. All plants were watered daily for 5 min with 1/1000 Hyponex solution (Hyponex, Osaka, Japan). At 37 days post-germination, plants were harvested within 2 hours from the end of the light period, in random order to minimize any variation due to harvest order. Samples were stored dry at –80°C before GC‒MS metabolomic profiling.

#### Time-course stress experiment

Time-resolved stress experiments using different light and temperature conditions were conducted in a previous study [36]. In brief, wild-type *A*. *thaliana* Col-0 was grown in soil (potting compost) in short days (8 h light) for 4 weeks, then transferred to long days (16 h light) at light/night temperature of 21/18°C for two weeks. Temperature- and light-stress treatments were conducted as follows: aside from the control condition (21°C and 150 μE m^–2^ sec^–1^, abbreviated as 21-L), the plants were exposed to seven different environmental conditions: (i) 4°C and darkness; (4-D), (ii) 21°C and darkness (21-D), (iii) 32°C and darkness (32-D), (iv) 4°C and 85 μE m^–2^ sec^–1^ (normal light; 4-L), (v) 21°C and 75 μE m^–2^ sec^–1^ (low light; 21-LL), (vi) 21°C and 300 μE m^–2^ sec^–1^ (high light; 21-HL), and (vii) 32°C and 150 μE m^–2^ sec^–1^ (normal light; 32-L). It should be noted that a reduced light intensity of 85 μE m^–2^ sec^–1^ was used in conjunction with the 4°C treatment in order to prevent a secondary stress caused by excess light [67]. The 4°C condition can therefore not be regarded as merely different in temperature compared to the 21-L or the 32-L conditions.

Plant material was sampled at 20 min intervals for a total of 360 min to yield a 19 data-point linear series (including 0 min). Additional samples were taken after 5, 10, 640, and 1280 min to obtain 10 data points (including 0 min) in a logarithmic time series. For each condition and each time point, three independent plants were sampled and analyzed for metabolites and transcripts.

#### Knockout mutant lines: selection, genotyping and growth conditions

*A*. *thaliana* Col-0 (wild-type) plants were used as control throughout the experiment. We obtained three SALK lines from the Arabidopsis Stock Center [68], with T-DNA insertions in the *AGT2* (*AT4G39660*; SALK_003381 and SALK_035035) and *HGO* (*AT5G54080*; SALK_027807) genes. Knockout lines were selected on plates supplemented with kanamycin, and non-segregating homozygous lines were genotyped. The following left primer (LP), right primer (RP) and border primer (BP) were designed using the Primer Design Tool provided by the Salk Institute Genomic Analysis Laboratory (http://signal.salk.edu/tdnaprimers.2.html) and used for the PCR analysis checking the presence of the T-DNA and zygosity in the offspring of the delivered seeds. For SALK_003381, LP (5’-TTTTGCTCTTGCATTAGTGGG-3’), RP (5’- CCTTCAACGATGTTAAGCTGC-3’), BP (5’-ATTTTGCCGATTTCGGAAC-3’); for SALK_035035, LP (5’-TACAGTGTCACTGTCGGTTGC-3’), RP (5’- CCTGCATCCAAATCATAGAGC-3’), BP (5’-ATTTTGCCGATTTCGGAAC-3’); for SALK_027807, LP (5’-GACAGGTGCTAATGGTCTTGC-3’), RP (5’- CAGCTTGGGTATTGAAAGTGG-3’), BP (5’-ATTTTGCCGATTTCGGAAC-3’) primers were used to test the lines. Quantitative PCR analysis of the mutant lines was performed with gene-specific primers (primer sequences: F 5’-AGTCACAATGGCAAAGGGAATTGG-3’ and R 5’-AGTCCACCAGCTGAACAAACCG-3’) for *AGT2*, and both of the T-DNA insertion mutants were shown to have complete knock-out of the gene. Regarding the AT5G54080 gene, we repeated the same RT-PCR analysis as previously described for analysis of the *hgo* mutant [69]. Two-week-old seedlings grown in MS were harvested. The *HGO* was amplified with primers (Forward: 5’- CGGTGAACTCTTTACTGCTA-3’ and Reverse: 5’-ATCTAAACCAACACCGTTAT-3’). PCR amplification conditions were as follows: 95°C for 2 min; 25 cycles of 94°C for 30 s, 51°C to 55°C for 30 s, and 72°C for 1 min; then 72°C for 10 min.

Knockout lines and control plants (Col-0) were grown, 12 biological replicates from each lines, in short-day condition for four weeks, then transferred to long-day condition for another two weeks. Next, we randomly divided the plants into two equal groups, one remaining in control untreated condition and the other exposed to stress (32-D) for continuous 1280 minutes, which mimics the stress condition in the time-course stress experiment. The rosettes of all plants in normal and stress conditions were harvested and frozen in liquid nitrogen, then stored at –80°C until subsequent GC‒MS measurement.

### Primary metabolite profiling by gas chromatography‒mass spectrometry

Metabolite extraction and derivatization from *A*. *thaliana* leaves using GC‒MS were performed as described by Lisec *et al* [70]. The GC‒MS data were obtained using an Agilent 7683 series auto-sample (Agilent Technologies, http://www.home.agilent.com), coupled to an Agilent 6890 gas-chromatograph‒Leco Pegasus two time-of-flight mass spectrometer (Leco; http://www.leco.com/). Identical chromatogram acquisition parameters were applied to those previously used [36]. Chromatograms were exported from LECO CHROMATOF software (version 3.34) to R software. Ion extraction, peak detection, retention time alignment and library searching were obtained using the TargetSearch package from Bioconductor [71]. Day-normalization and sample median-normalization were conducted; the resulting data matrix was used for further analysis.

### Genome-wide associations

#### Data acquisition for GWAS and mapping

200K SNP data for 314 *A*. *thaliana* accessions, obtained using Affymetrix GeneChip Array 6.0, were taken from previous publications [65, 66]. Metabolic profiling was performed using GC‒MS as described above. In order to avoid spurious false positive associations due to small sample sizes, only metabolic traits with non-missing values across at least 40% of the accession samples were included in the data preprocessing. Following this initial quality control, 94 primary metabolites were detected. Metabolite concentrations were log-transformed since a test of normality showed that in most cases the log-transformed concentrations were closer to a normal distribution than the non-transformed values [72]. Genome-wide association analysis for metabolite traits was performed using 199,455 SNPs with minor allele frequency > 1% across 314 accessions to investigate the associations between metabolite traits and SNPs. At each of these SNPs, a compressed mixed linear model [73] was fitted for each trait in the Genome Association and Prediction Integrated Tool (GAPIT) R package [74]. This model includes principal components as fixed effects to account for population structure (commonly called the “Q” matrix) [37], and a kinship matrix (commonly called the “K” matrix) [38] to account for family relatedness across the accessions. The SNP fraction parameter was set to 0.1 to avoid excessive computation, as recommended by the GAPIT user manual. Other parameters were set as default values.

#### Locus identification

The following procedure was applied to identify genomic regions associated with the metabolite traits. First, we extracted all SNPs displaying a Bonferroni corrected *p*-value < 0.05 in any of the 94 primary metabolites. Then all the SNPs with logarithm of odds (LOD) value >–log_10_ (1/*N*) (*N* is the number of SNPs used in the study) were extracted as described previously [20]. LOD threshold was set as 5.3 by using this method. The resulting SNPs were assigned to the same group if the genomic distance between them was less than 10 kb. For each SNP group, we kept those that had at least one SNP with Bonferroni corrected *p*-value < 0.05, and the rest of the groups were discarded. Finally, all the genes around or within the resulting groups were taken into account as putative candidates.

### Network analysis

#### Transcript and metabolite data acquisition from time-course stress experiments

Transcript and metabolite data from time-course stress experiments were derived from previous work [36], resulting in 15,089 transcripts and 92 primary metabolites (including 27 unknown primary metabolites) for further analysis. Significantly changed primary metabolites across 23 time points in each condition were selected by ANOVA using “aov” function in R (http://www.r-project.org/) at a significance level of 0.05 with a multiple correction test using false discovery rate (FDR) estimation [75] by comparing three replicates at all time points. All 15,089 transcripts and the metabolites that changed significantly in each condition were used for the construction of condition-specific networks.

#### Condition-specific network construction

Based on transcript and metabolite data from the dense time-course experiment under eight conditions, Pearson correlation coefficient (PCC) between metabolite and transcript features was calculated in R. We chose PCC as correlation measure in this study for two reasons: (1) PCC is the most widely used correlation measure [76], and it provides more accurate results because it is a parametric measure [77]; (2) it was found that the statistical significances of correlation coefficients obtained though parametric and non-parametric methods were compatible in 95% of the cases when combining metabolomics and transcriptomics data [77]. PCC thresholds for building edges between features (metabolites and transcripts) in networks in each condition were obtained based on a permutation test (FDR < 0.05). Undirected networks for each condition were constructed with nodes representing metabolite and transcript features and edges connecting the nodes between features with a PCC passing the threshold using the igraph package [78] in R.

### Comparison of the performance of individual and combined methods

#### Procedure setup for determining method performance

The performance of each single method (GWAS, RIL, IL, and network analysis) and of the combined strategy (network analysis and at least one quantitative genetics approach) was tested by *precision*, *recall* and *F*-measure.

*Reference Gene List (RGL) generation*: we first built four reference gene lists (RGL1, RGL2, RGL3 and RGL4; **S7 Table**) for all the metabolites shown in the different datasets based on KEGG metabolic pathway [50]. The reference gene lists are classified according to the layers of enzymes in chemical reactions surrounding a given metabolite in an increasing order as follow:

1. RGL1: enzymes involved in the direct catalytic reactions of a given metabolite (step = 1)
2. RGL2: enzymes at a distance of two (or less) steps of catalytic reactions from a given metabolite (step ≤ 2)
3. RGL3: enzymes at a distance of three (or less) steps of catalytic reactions from a given metabolite (step ≤ 3)
4. RGL4: all the enzymes in the pathways that a given metabolite is involved in

*Actual gene list generation*: next, we generated the actual gene lists for each metabolite in each dataset. Separate lists of all the genes identified in the mQTL for each metabolite trait in each of the mapping populations (GWAS, RIL, and IL) were generated; in parallel, lists of genes based on neighbor transcripts in the networks for each metabolite were extracted. For the combined strategy, we set up a combined gene list comprising the genes that are shared between the network approach and at least one of the quantitative genetics approaches for each metabolite.

*Precision*, *recall and F-measure calculation*: all the above-mentioned actual gene lists for each metabolite were compared with the four reference gene lists (RGL1–RGL4). We considered the metabolites to be correctly predicted if at least one gene could be matched between the actual gene list and the reference gene lists. The parameter *precision* represents the positive predictive value of the method; *recall* is equivalent to sensitivity. The two metrics are often combined as their harmonic mean, known as the *F-measure*. The performance of the different methods can be assessed by the above-mentioned three statistics:

$$precision=\frac{N_{cp}}{N_{p}}$$

$$recall=\frac{N_{cp}}{N_{a}}$$

$$F-measure=\frac{2\times precision\times recall}{precision+recall}$$

Where *N*_*a*_ is the number of all annotated metabolites in each dataset, *N*_*p*_ is the number of relevant metabolites that have mQTL in mapping approaches or neighbor transcripts in network analysis, and *N*_*cp*_ is the number of annotated metabolites that can be correctly predicted when comparing with the reference gene lists.

*Metabolite-wise precision calculation*: another parameter, *metabolite-wise precision* [for a certain metabolite: the number of correctly predicted genes is divided by the number of all the genes in the respective mQTL (in mapping approaches) or is divided by the number of all the neighbor transcripts around this metabolite (in network analysis)], was also calculated in order to show the percentage of true positively discovered genes for each metabolite.

#### Testing GWAS performance based on different LOD thresholds and LOD threshold optimization for data integration

To determine the optimal GWAS LOD threshold for integration with other methods, *precision*, *recall* and *F-measure* were calculated for the GWAS dataset based on different GWAS LOD thresholds ranging from Bonferroni corrected significant threshold 5.3 to 2.0 (*cf*. **S8 Table**). In addition, for each annotated metabolite in the GWAS dataset, the *metabolite-wise precision* was calculated using various LOD thresholds (*cf*. **S2 Fig**).

#### Performance comparison for different methods

In order to compare the performance of the different methods, *precision*, *recall* and *F-measure* were calculated for each individual method and for the combined strategy based on LOD thresholds ranging from 3.0 to 5.3 (*cf*. **S4** and **S5 Figs** and **Fig 5**).

Additionally, the *metabolite-wise precision* was compared between every single method and the combined strategy based on both LOD thresholds (5.3 and 4.5) by using ANOVA. Subsequently, pair-wise comparison was conducted by the Tukey HSD tests using the “TukeyHSD” function in R (*cf*. **S6** and **S7 Figs**).

#### Permutation test for the combined strategy

The performance of the combined strategy was further evaluated by permutation test using the same number of randomly selected genes for each metabolite. To estimate a *p*-value empirically, we shuffled the related genes for all the annotated metabolites in the combined dataset, then compared with the four reference gene lists to obtain the permuted *F-measure* value. We then compared the true *F-measure* (*x*) and permuted *F-measure* (*y*_*k*_) in *k* permutations (*k* = 10,000):

$$p=\frac{1}{n}\;\sum_{k=1}^{n}F(x,y_{k})$$

$$F(x,y)=\left\{ \begin{array}{l} 0\;for\;x>y_{k}\\ 1\;else \end{array}\right.$$

Hence, if the true *F-measure* is higher than the permuted *F-measure* for 950 of the 1000 permutations, we obtain a *p*-value estimate of 0.05.

### Statistics for knockout validation experiment

Metabolite intensity data after transformation and normalization were used for ANOVA to test the significance levels of metabolite changes in knockout and Col-0 plants under normal and stress conditions, following by correction for multiple comparisons using the “p.adjust” function in R (http://www.r-project.org/). Subsequently, pair-wise comparison was conducted by the Tukey HSD tests using the “TukeyHSD” function in R.

### Phylogenetic analysis

Target *A*. *thaliana* protein sequences in this study were extracted from The Arabidopsis Information Resource (TAIR, https://www.arabidopsis.org/). The sequences of all biochemically characterized alanine aminotransferases and AGT-like proteins from other species were extracted from NCBI (http://www.ncbi.nlm.nih.gov/) and PLAZA 3.0 (http://bioinformatics.psb.ugent.be/plaza/). Amino-acid sequences were aligned using the CLUSTALW (version 1.83) program. A maximum likelihood tree was constructed using MEGA 7.0 software with all default parameters.

## Supporting Information

S1 Dataset Normalized intensities of 94 primary metabolites in 314 accessions of *A*. *thaliana* germplasms. (XLSX)

S1 Fig Quantile–quantile (QQ) plots of *p*-value for eleven metabolite traits. The Y-axis is the observed negative base 10 logarithm of the *p*-values; the X-axis is the expected observed negative base 10 logarithm of the *p*-values under the assumption that the *p*-values follow a uniform [0, 1] distribution. The dotted lines show the 95% confidence interval for the QQ plot under the null hypothesis of no association between the SNP and the trait.
(TIF)

S2 Fig *Metabolite-wise precision* test for different LOD thresholds ranging from 3.0 to 5.3 based on the four reference gene lists. The X-axis shows the different LOD thresholds ranging from 3.0 to 5.3; the Y-axis shows *metabolite-wise precision* values.
(TIF)

S3 Fig Tyrosine and *TAT7* in an association detected by network analysis and RIL mapping. The reaction catalyzed by the tyrosine aminotransferase TAT7 (AT5G53970) (upper panel). Time-resolved profiles of tyrosine and L-glutamate levels and *TAT7* expression levels under five different conditions (bottom panel). Data adapted from [36].
(TIF)

S4 Fig *Precision* comparison between the single methods (GWAS, RIL, IL, and network analysis) and the combined strategy using different LOD thresholds based on the four reference gene lists. (TIF)

S5 Fig *Recall* comparison between the single methods (GWAS, RIL, IL, and network analysis) and the combined strategy using different LOD thresholds based on the four reference gene lists. (TIF)

S6 Fig *Metabolite-wise precision* comparison between the single methods (GWAS, RIL, IL, and network analysis) and the combined strategy using LOD threshold 5.3 based on the four reference gene lists. (TIF)

S7 Fig *Metabolite-wise precision* comparison between the single methods (GWAS, RIL, IL, and network analysis) and the combined strategy using LOD threshold 4.5 based on the four reference gene lists. (TIF)

S8 Fig Tyrosine profiles in eight conditions based on the time-course stress experiment previously described in [36]. (TIF)

S9 Fig Phylogenetic analysis of the *AGT2* gene family in *A*. *thaliana* and in ten other species. The maximum likelihood tree was constructed using aligned full-length amino-acid sequences. Bootstrap values from 1,000 replicates are indicated at each node. Bar = 0.1 amino-acid substitutions per site. The following gene sequences were used for the analysis: AT2G38400, AT3G08860, AT4G39660 (*A*. *thaliana*); AL3G08940, AL4G24720, AL7G01820 (*A*. *lyrata*); BR01G00590, BR03G19080, BR03G31870, BR08G19740 (*Brassica rapa)*; GM18G02440, GM05G31630, GM08G14850 (*Glycine max*); GR05G00650, GR06G23360, GR11G13020 (*Gossypium raimondii*); MT3G465800, MT7G072420, MT8G091660 (*Medicago truncatula*); OS03G07570, OS03G21960, OS05G39770 (*Oryza Sativa* ssp. *japonica*); SL04G054310, SL10G076250 (*Solanum lycopersicum*); ST04G022360, ST10G018540 (*S*. *tuberosum*); VV06G00800, VV08G06170, VV07G06090 (*Vitis vinifera*); ZM01G05170, ZM06G26480, ZM06G26540 (*Zea mays*). The target gene (AT4G39660) in this study is highlighted with a red box, and the homolog in maize (ZM01G05170) is highlighted with a blue diamond.
(TIF)

S10 Fig β-alanine profiles in eight conditions based on the time-course stress experiment previously described in [36]. (TIF)

S1 Table List of 314 *A*. *thaliana* accessions and related information. (XLSX)

S2 Table Summary of metabolite information including metabolite ID, metabolite name, metabolite class, and mass used for quantification in the genome-wide association study. (XLSX)

S3 Table Full list of significant associations between SNPs and primary metabolites with *p*-value < 1/N (N is the number of SNP markers used in this study). MAF: minor allele frequency.
(XLSX)

S4 Table Full list of significant associations between genes and primary metabolites with *p*-value < 1/N (N is the number of SNP markers used in this study). (XLSX)

S5 Table Metabolite traits in the RIL dataset and 32 common metabolite traits in comparison with the GWAS dataset. (XLSX)

S6 Table Summarized QTL results for introgression line dataset. (XLSX)

S7 Table Four reference gene lists (RGL1, RGL2, RGL3, and RGL4) for all the metabolites shown in the different datasets based on KEGG metabolic pathway. (XLSX)

S8 Table GWAS performance based on different LOD thresholds ranging from 2.0 to 5.3. (XLSX)

S9 Table Common loci verified by GWAS, RIL and IL datasets using the suggested LOD threshold of 4.4. (XLSX)

S10 Table Numbers of metabolites and transcripts, and related PCC thresholds obtained by permutation test (*p* = 0.05) for network analysis. (XLSX)

S11 Table Information of metabolites, transcripts, correlation values in 219 conserved associations shared in all 8 conditions. (XLSX)

S12 Table List of common metabolite-transcript associations between GWAS and network analysis using the strict LOD threshold (LOD = 5.3). (XLSX)

S13 Table List of common transcript-metabolite associations between GWAS and network analysis using the suggested LOD threshold (LOD = 4.5). (XLSX)

S14 Table Information on the associations supported by GWAS, network analysis, RIL and IL datasets based on the strict LOD threshold 5.3. (XLSX)

S15 Table Information on the associations supported by GWAS, network analysis, RIL and IL datasets based on the suggested LOD threshold 4.5. (XLSX)
