# Supplementary figures and images for: Combined Use of Genome-Wide Association Data and Correlation Networks Unravels Key Regulators of Primary Metabolism in Arabidopsis thaliana
Source: PLoS Genet. 2016 Oct 19;12(10):e1006363. doi: 10.1371/journal.pgen.1006363 (PMC5070769; doi:10.1371/journal.pgen.1006363)

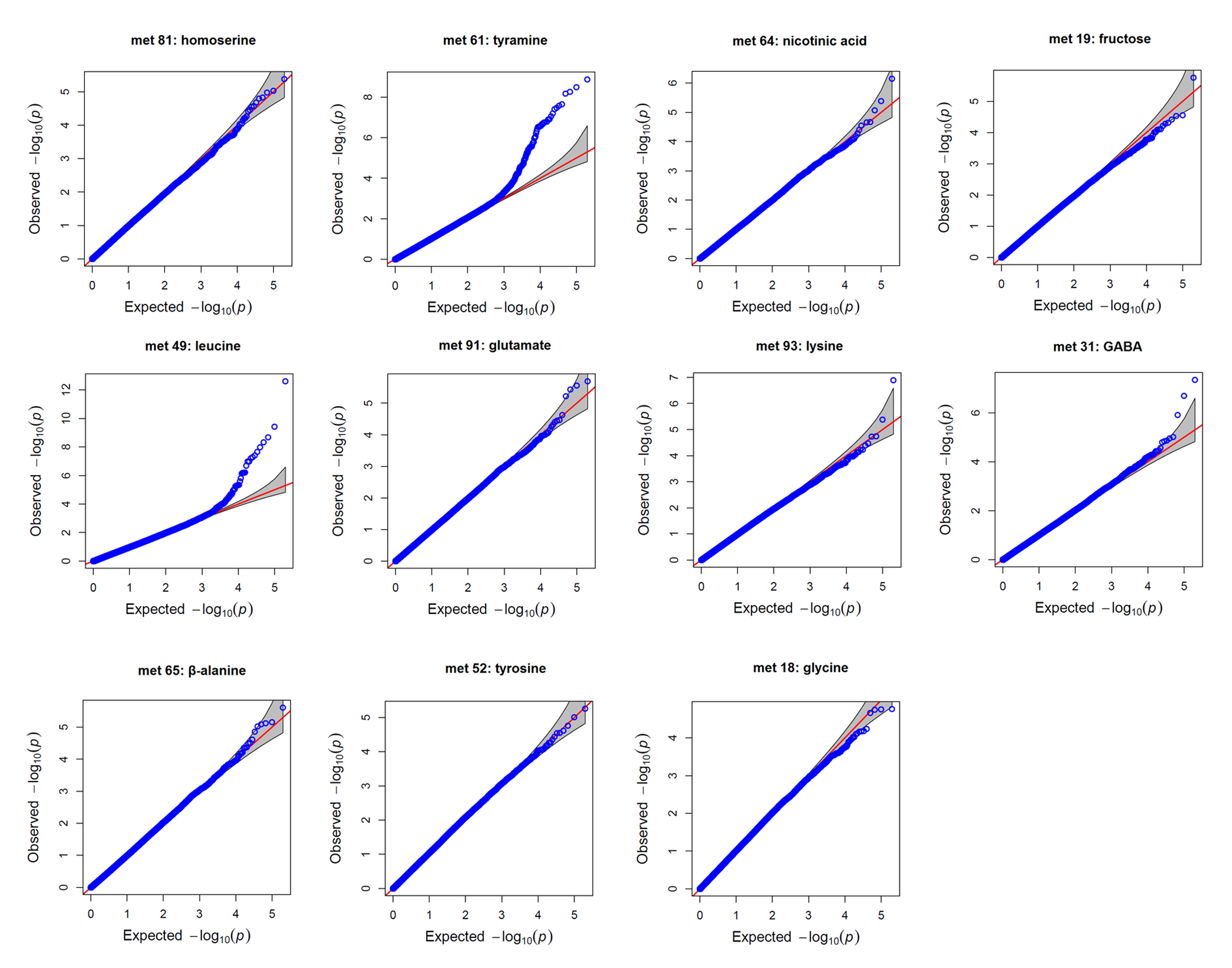

Supplement: S1 Fig — The Y-axis is the observed negative base 10 logarithm of the p-values; the X-axis is the expected observed negative base 10 logarithm of the p-values under the assumption that the p-values follow a uniform [0, 1] distribution. The dotted lines show the 95% confidence interval for the QQ plot under the null hypothesis of no association between the SNP and the trait. (TIF) [file pgen.1006363.s002.tif]

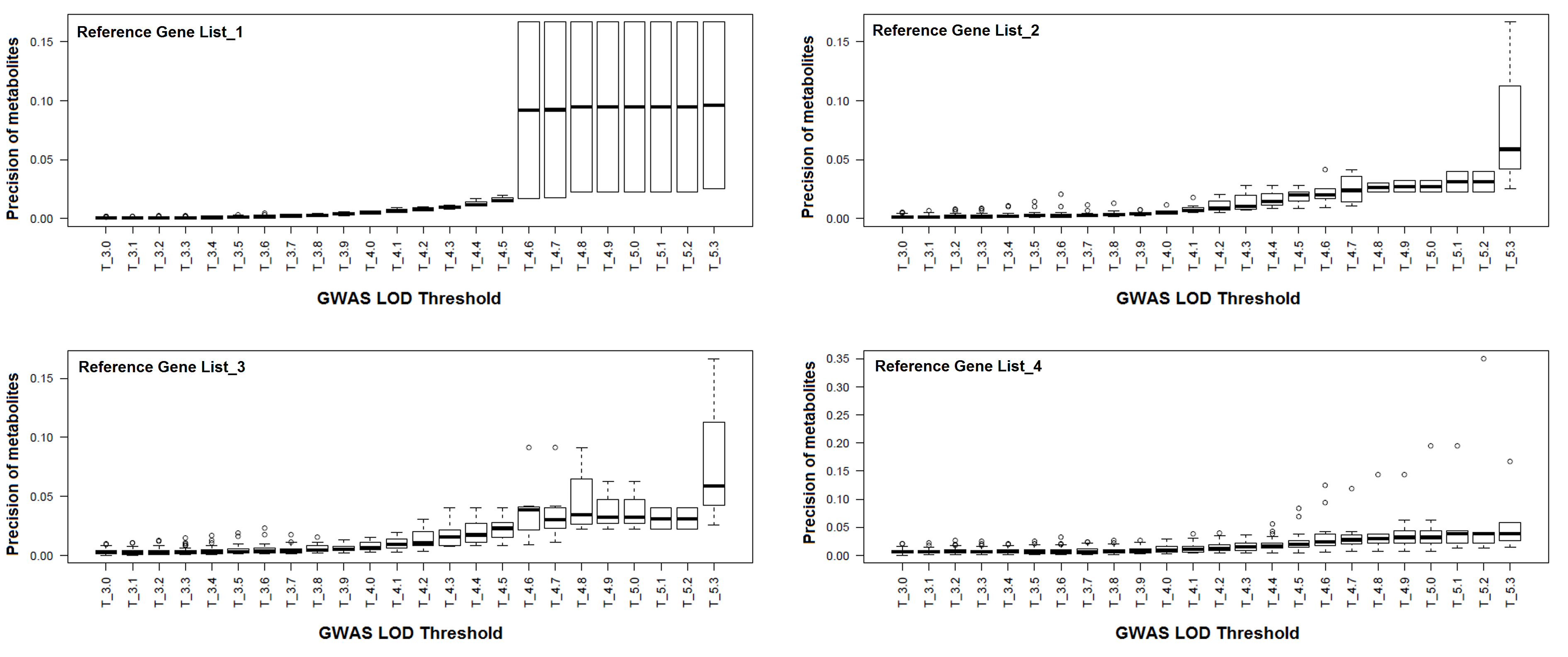

Supplement: S2 Fig — The X-axis shows the different LOD thresholds ranging from 3.0 to 5.3; the Y-axis shows metabolite-wise precision values. (TIF) [file pgen.1006363.s003.tif]

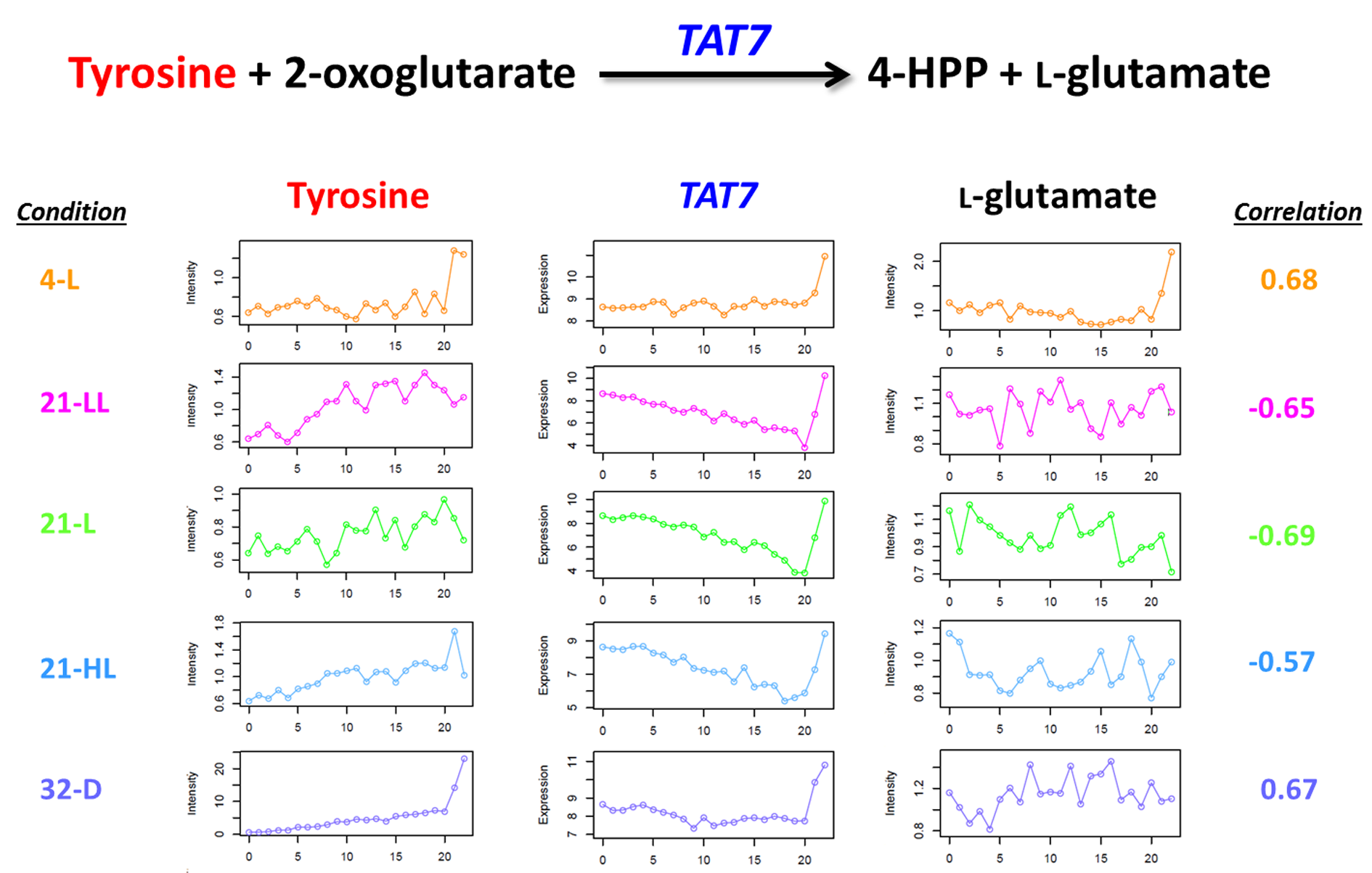

Supplement: S3 Fig — The reaction catalyzed by the tyrosine aminotransferase TAT7 (AT5G53970) (upper panel). Time-resolved profiles of tyrosine and L-glutamate levels and TAT7 expression levels under five different conditions (bottom panel). Data adapted from [36]. (TIF) [file pgen.1006363.s004.tif]

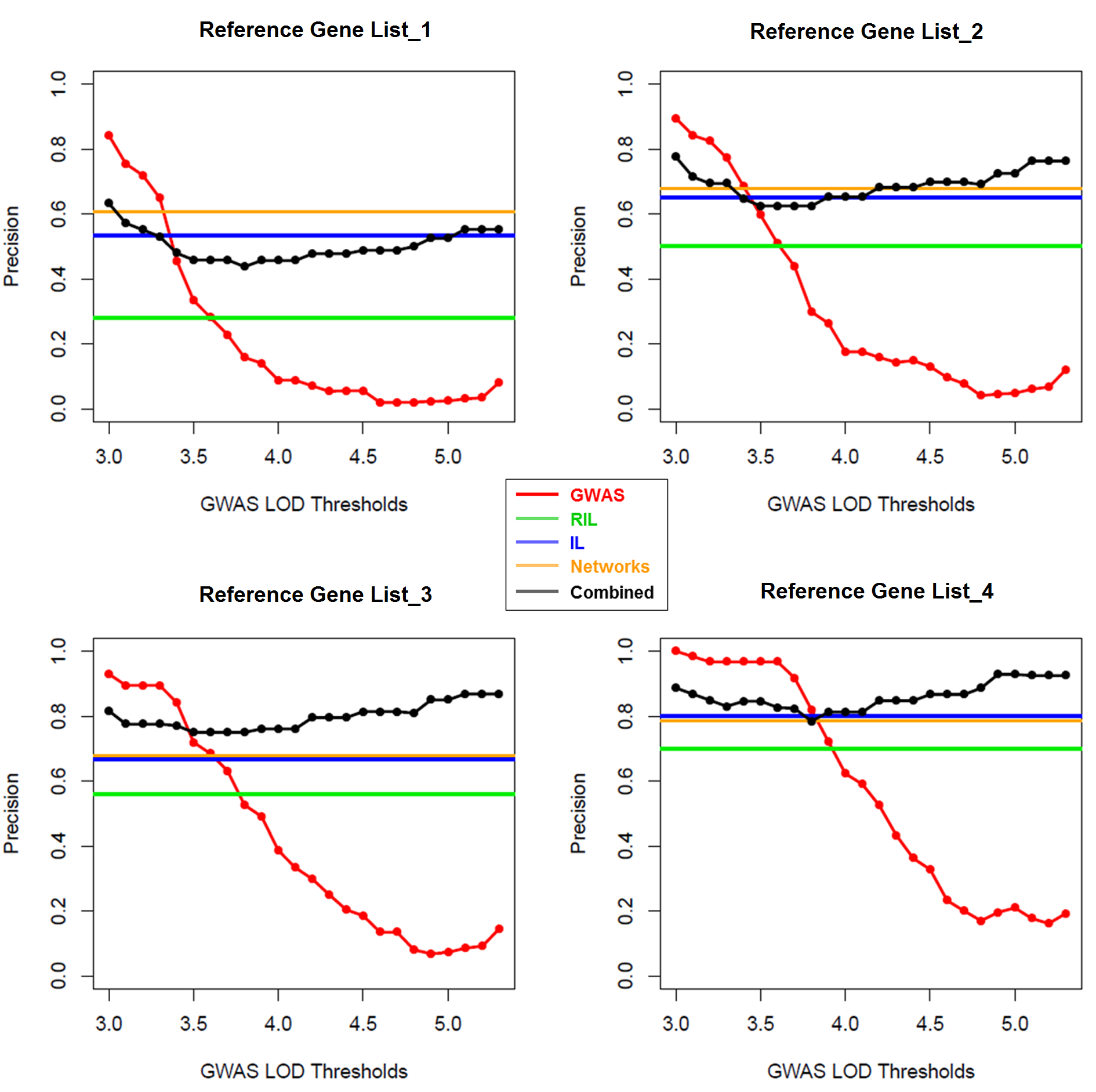

Supplement: S4 Fig — (TIF) [file pgen.1006363.s005.tif]

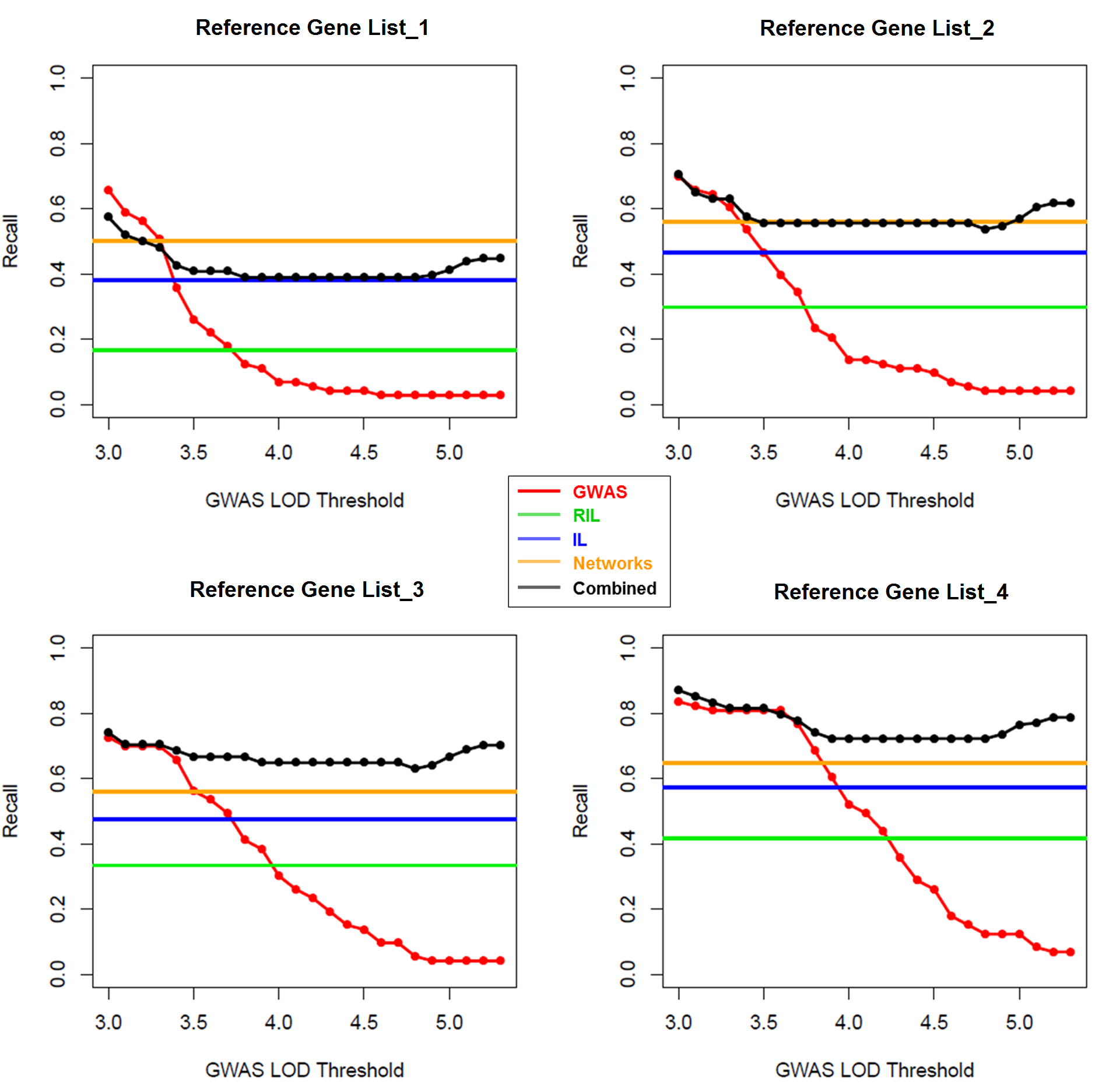

Supplement: S5 Fig — (TIF) [file pgen.1006363.s006.tif]

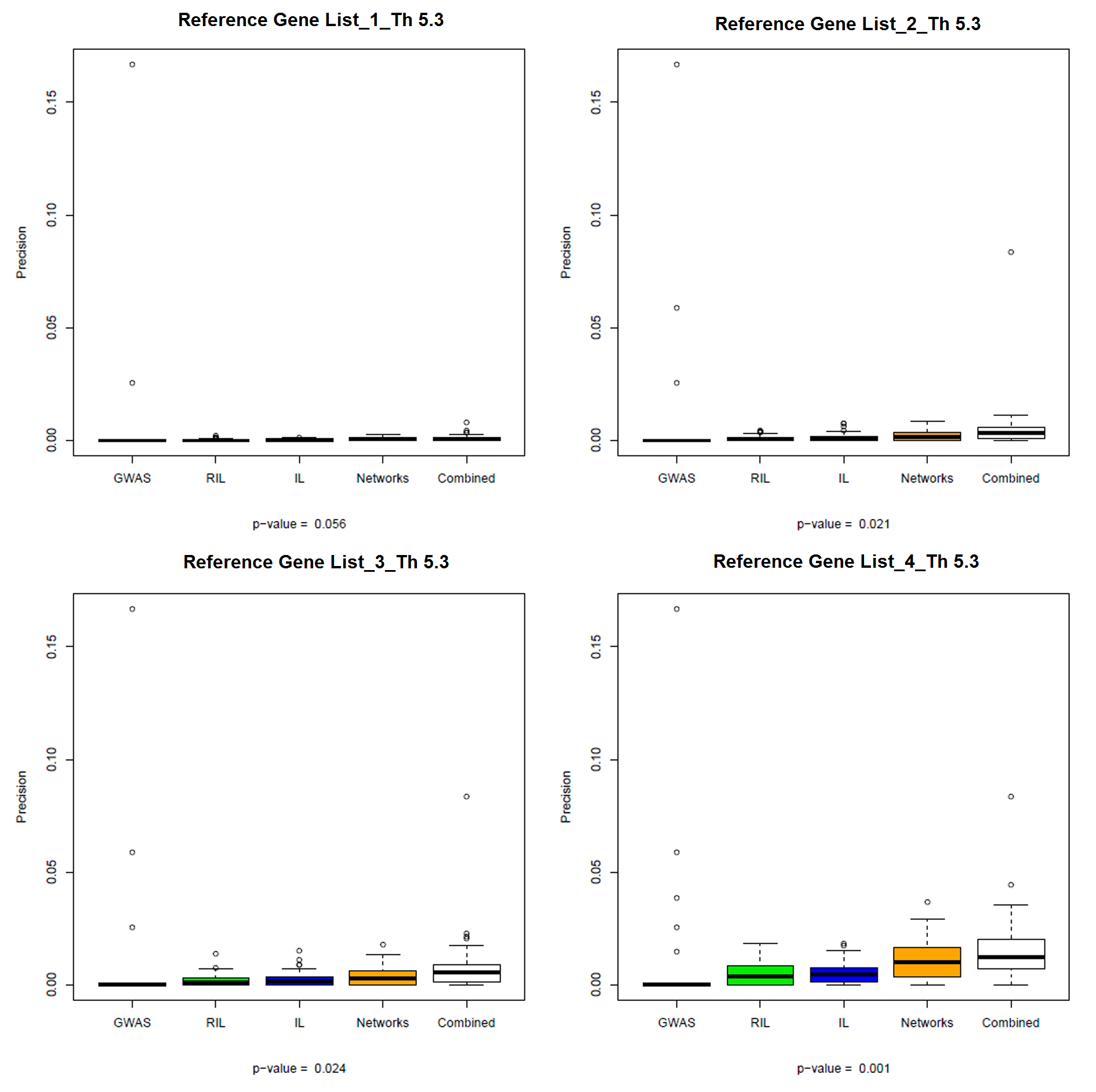

Supplement: S6 Fig — (TIF) [file pgen.1006363.s007.tif]

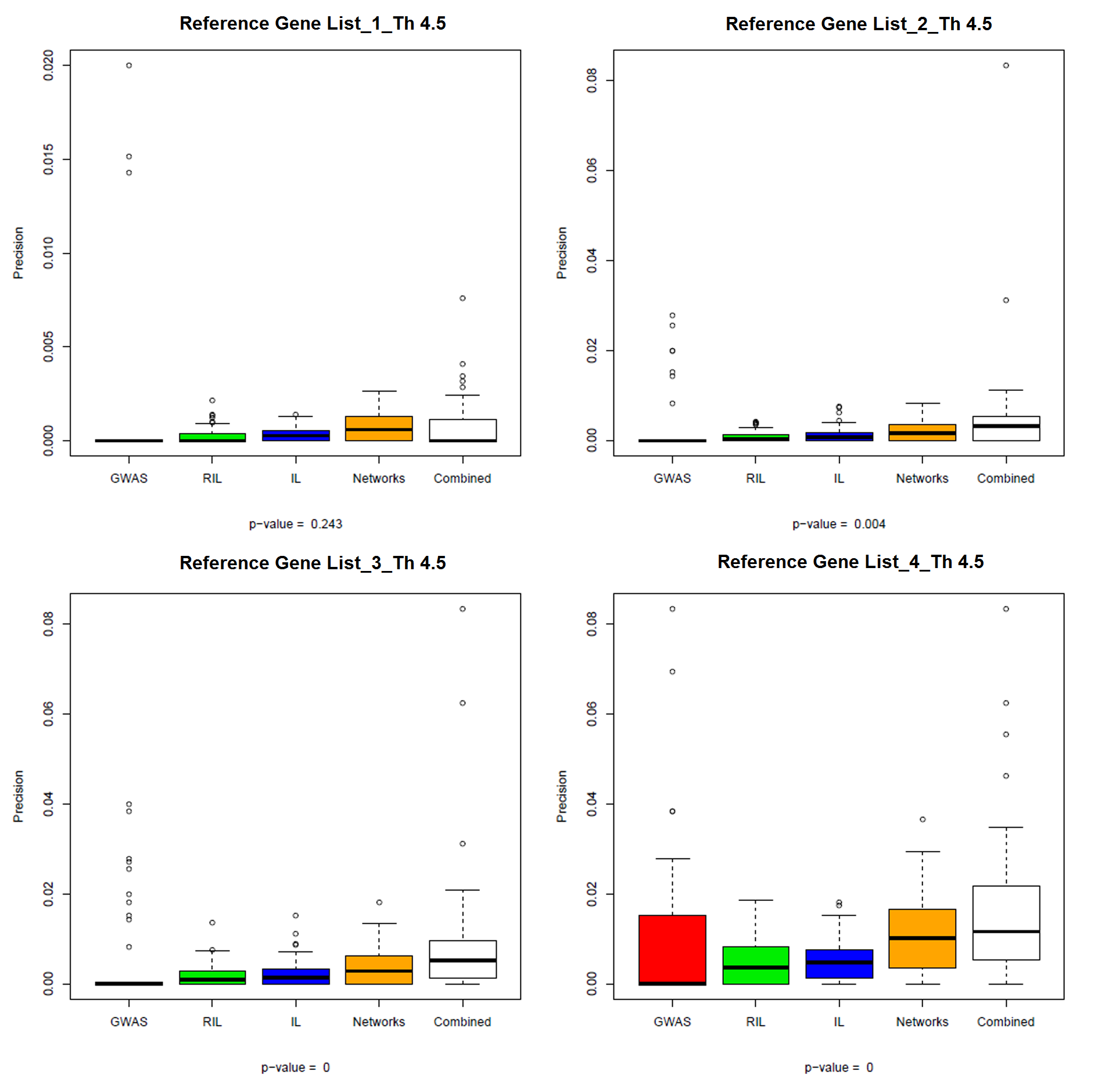

Supplement: S7 Fig — (TIF) [file pgen.1006363.s008.tif]

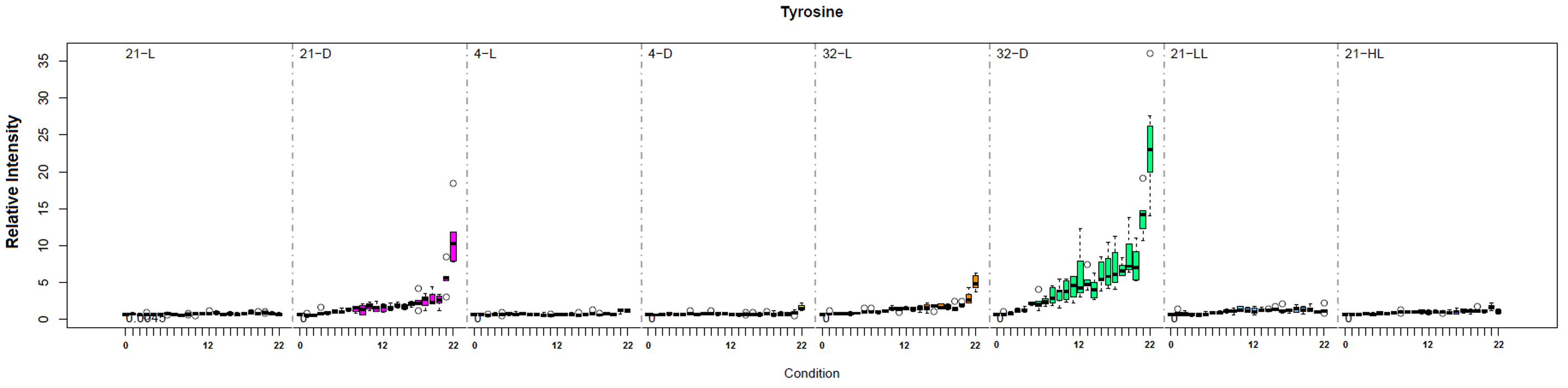

Supplement: S8 Fig — (TIF) [file pgen.1006363.s009.tif]

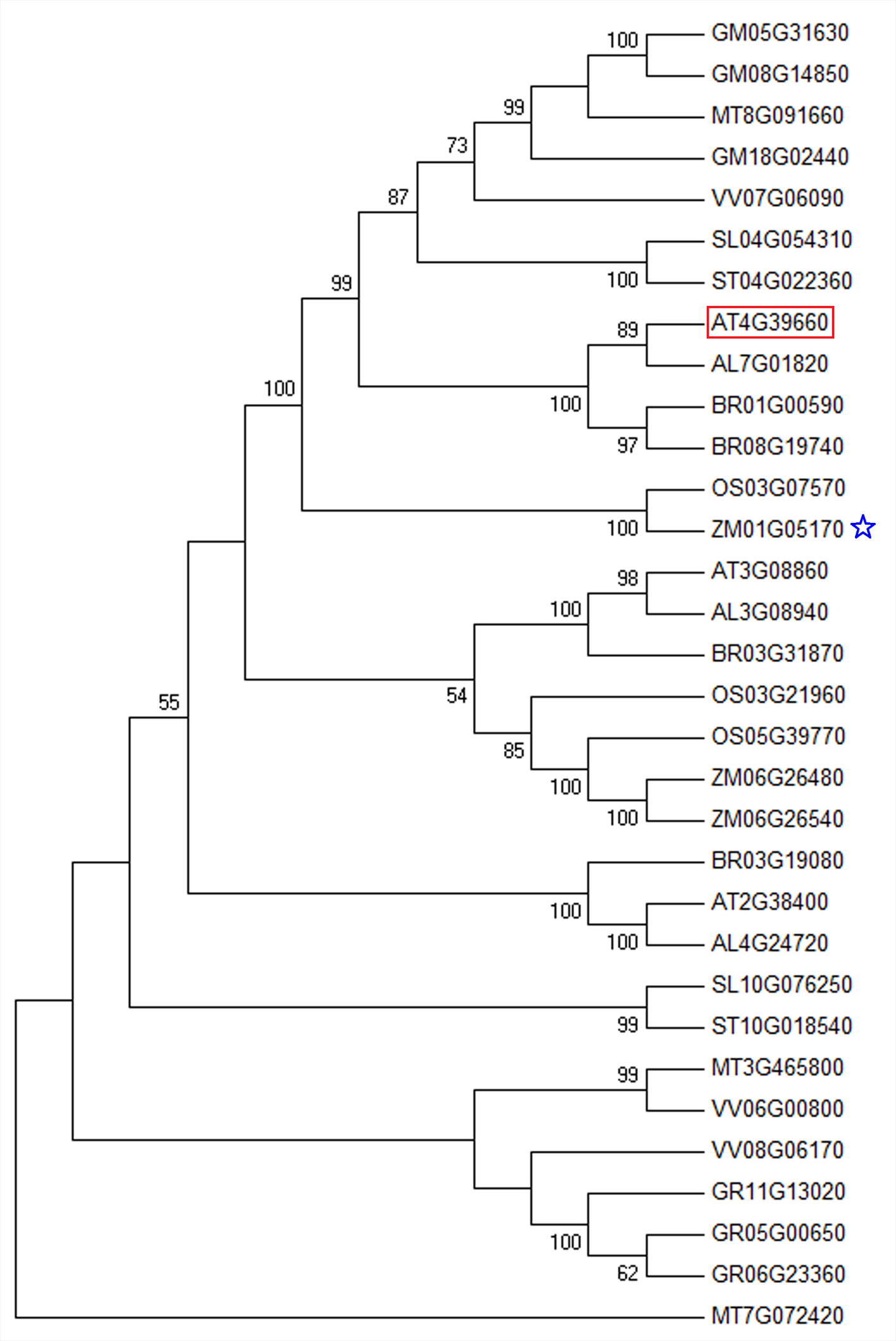

Supplement: S9 Fig — The maximum likelihood tree was constructed using aligned full-length amino-acid sequences. Bootstrap values from 1,000 replicates are indicated at each node. Bar = 0.1 amino-acid substitutions per site. The following gene sequences were used for the analysis: AT2G38400, AT3G08860, AT4G39660 (A. thaliana); AL3G08940, AL4G24720, AL7G01820 (A. lyrata); BR01G00590, BR03G19080, BR03G31870, BR08G19740 (Brassica rapa); GM18G02440, GM05G31630, GM08G14850 (Glycine max); GR05G00650, GR06G23360, GR11G13020 (Gossypium raimondii); MT3G465800, MT7G072420, MT8G091660 (Medicago truncatula); OS03G07570, OS03G21960, OS05G39770 (Oryza Sativa ssp. japonica); SL04G054310, SL10G076250 (Solanum lycopersicum); ST04G022360, ST10G018540 (S. tuberosum); VV06G00800, VV08G06170, VV07G06090 (Vitis vinifera); ZM01G05170, ZM06G26480, ZM06G26540 (Zea mays). The target gene (AT4G39660) in this study is highlighted with a red box, and the homolog in maize (ZM01G05170) is highlighted with a blue diamond. (TIF) [file pgen.1006363.s010.tif]

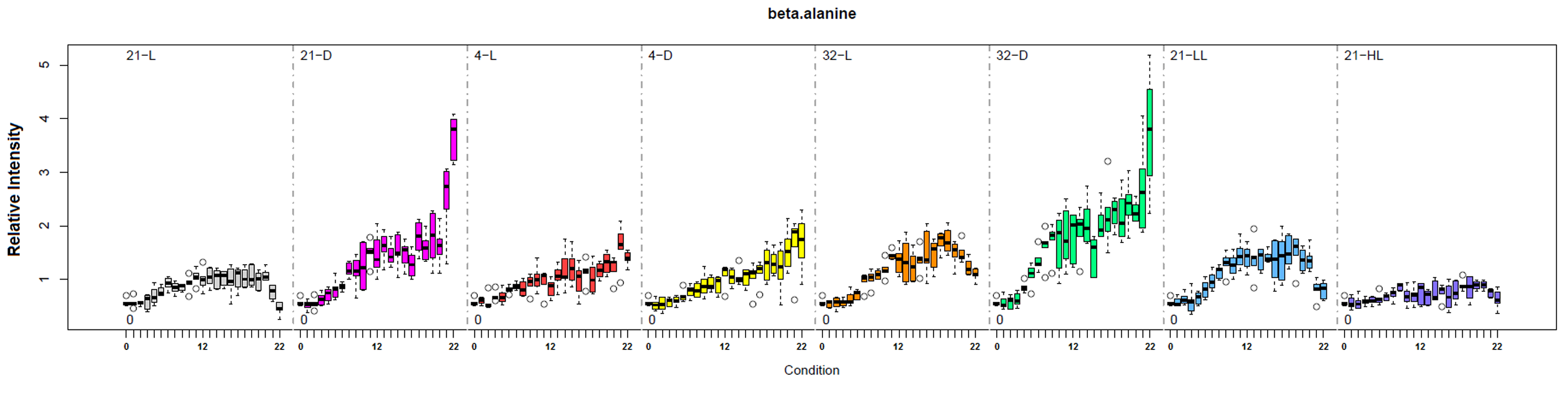

Supplement: S10 Fig — (TIF) [file pgen.1006363.s011.tif]
